# Linear polyubiquitylation of Gli protein regulates its protein stability and facilitates tumor growth in colorectal cancer

**DOI:** 10.1038/s41420-024-02147-4

**Published:** 2024-08-20

**Authors:** Junyao Cheng, Linlin Xu, Yanlu Xuan, Feifei Zhou, Aidi Huang, Shaopeng Zeng, Hailong Wang, Yiting Wang, Yuan Zhan, Xiaohua Yan, Shiwen Luo, Yuan Liu, Minzhang Cheng

**Affiliations:** 1 Center for Experimental Medicine, The MOE Basic Research and Innovation Center for the Targeted Therapeutics of Solid Tumors, the First Affiliated Hospital, Jiangxi Medical College, Nanchang University, Nanchang, Jiangxi China; 2 Jiangxi Provincial Key Laboratory of Respiratory Diseases, Jiangxi Institute of Respiratory Diseases, The Department of Respiratory and Critical Care Medicine, the First Affiliated Hospital, Jiangxi Medical College, Nanchang University, Nanchang, Jiangxi China; 3 Jiangxi Provincial Key Laboratory for Precision Pathology and Intelligent Diagnosis, Department of Pathology and Institute of Molecular Pathology, the First Affiliated Hospital, Jiangxi Medical College, Nanchang University, Nanchang, Jiangxi China; 4 Department of Gastroenterology, Shandong Provincial Hospital Affiliated to Shandong First Medical University, Jinan, Shandong China; 5 Medical Innovation Centre, the First Affiliated Hospital, Jiangxi Medical College, Nanchang University, Nanchang, Jiangxi China; 6 Department of Oncology, the First Affiliated Hospital, Jiangxi Medical College, Nanchang University, Nanchang, Jiangxi China; 7 The MOE Basic Research and Innovation Center for the Targeted Therapeutics of Solid Tumors, School of Basic Medical Sciences, Jiangxi Medical College, Nanchang University, Nanchang, Jiangxi China; 8 The State Key Laboratory of Membrane Biology, Tsinghua-Peking Center for Life Sciences, School of Life Sciences, Tsinghua University, Beijing, China

**Keywords:** Colorectal cancer, Cell signalling

## Abstract

The linear ubiquitin chain assembly complex (LUBAC) mediates the linear ubiquitination of various proteins and is involved in NF-κB signaling and immune regulation. However, the function and mechanism of linear ubiquitination in regulating oncogenic signaling and tumor growth have remained poorly understood. Herein, we identified Gli proteins, key transcription factors in the Hedgehog (Hh) signaling pathway, as novel substrates of LUBAC. Linear ubiquitination stabilizes Gli proteins, leading to the noncanonical activation of Hh signaling in CRC cells. Furthermore, LUBAC facilitates tumor growth in CRC cells. Additionally, elevated expression of LUBAC components in CRC tissues was observed, and higher expression levels of these components correlated with poor prognosis in CRC patients. Interestingly, inhibition of LUBAC using either a small molecule agonist or RNA silencing specifically suppressed cell growth in CRC cells but had no effect on normal intestinal cells. Taken together, aberrant expression of LUBAC components activates Hh signaling noncanonically by mediating linear ubiquitination, promoting tumor growth in CRC, demonstrating the novel function of linear ubiquitination in regulating the protein stability of its substrates and highlighting the potential of targeting LUBAC as a therapeutic strategy in CRC.

## Introduction

Ubiquitination is one of the most ubiquitous and important post-translational modifications of proteins in cells and marks their substrates for degradation or other signaling, therefore regulating multiple biological processes [1]. Generally, ubiquitin is polymerized via its seven surface lysine residues (K6, K11, K27, K29, K48 and K63), whereas the linear ubiquitin chain, also called the M1-ubiquitin chain, is linked through the N-terminal methionine residue (M1) of ubiquitin [2].

Although linear ubiquitin chain assembly complex (LUBAC) has been identified as the only E3 ligase complex mediating linear ubiquitination modification since 2006 [3], knowledge about its function besides mediating TNF signaling and regulating autoimmunity has been limited for years [2]. Recently, many potential substrates of linear polyubiquitination have been identified via high-throughput screening by mass spectrometry [4] or proteome microarray [5]. Its cellular functions are now known to include regulation of mitosis [6], autophagy [7], and ciliogenesis [8], and its roles in governing angiogenesis [9, 10] and haematopoiesis [11] have been identified in embryonic development.

Although many reports have identified the function of each component of LUBAC individually, how linear ubiquitination participates in tumors remains unclear. Early research found that HOIP mediates monoubiquitylation of p53 and decreases its stability, therefore facilitating drug resistance in breast cancer cells [12]. Sharpin was reported to promote Wnt signaling by stabilizing β-catenin independently of linear ubiquitination in gastric cancer [13]. Another component of LUBAC, HOIL-1, also facilitates tumor growth in breast cancer by stabilizing oestrogen receptor α (ERα), but whether linear ubiquitination participates in this process remains unclear [14]. Recently, PTEN was identified as a novel linear polyubiquitylation substrate in prostate cancer, impairing its function and promoting prostate cancer progression [15].

Hedgehog (Hh) signaling is essential for cell proliferation and differentiation in embryonic development, and its dysregulation in embryos often results in multiple developmental defects [16]. Hh signaling is initiated by Hh ligands, including Sonic hedgehog (Shh), Indian hedgehog. (Ihh), or Desert hedgehog (Dhh), followed by activation of its downstream cascade, which comprises Patched homologue (PTCH), Smoothened homologue (SMO), and the family of Gli transcription factors. Among the three Gli homologues, Gli2 and Gli3 can undergo partial proteolysis to generate a suppressive form in the absence of Hh ligands, while Gli1 lacks this suppressive domain [17].

Because of its key roles in cell fate determination and cell proliferation, hyperactivation of Hh signaling promotes tumorigenesis and cancer progression in a variety of tumors, such as medulloblastoma [18], hepatocellular carcinoma [19], gastric cancer [20] and colorectal cancer [21]. Therefore, Hh signaling is tightly regulated by negative feedback mechanisms; in particular, the protein stability of Gli proteins is precisely governed by multiple ubiquitin E3 ligases [22, 23] and deubiquitinates [24, 25] through ubiquitination-dependent protein degradation.

Herein, we identified Gli proteins as novel substrates of LUBAC. Linear polyubiquitylation stabilizes Gli2 and Gli3 and therefore activates Hh signaling in CRC cells. Moreover, components of LUBAC were highly expressed in CRC tissues, and their higher expression was associated with poor prognosis in CRC patients. Our findings suggest LUBAC and linear ubiquitylation as potential therapeutic targets to restore Gli protein levels and suppress tumor growth in CRC.

## Results

### HOIP is highly expressed in CRC tissues

**Fig. 1:**
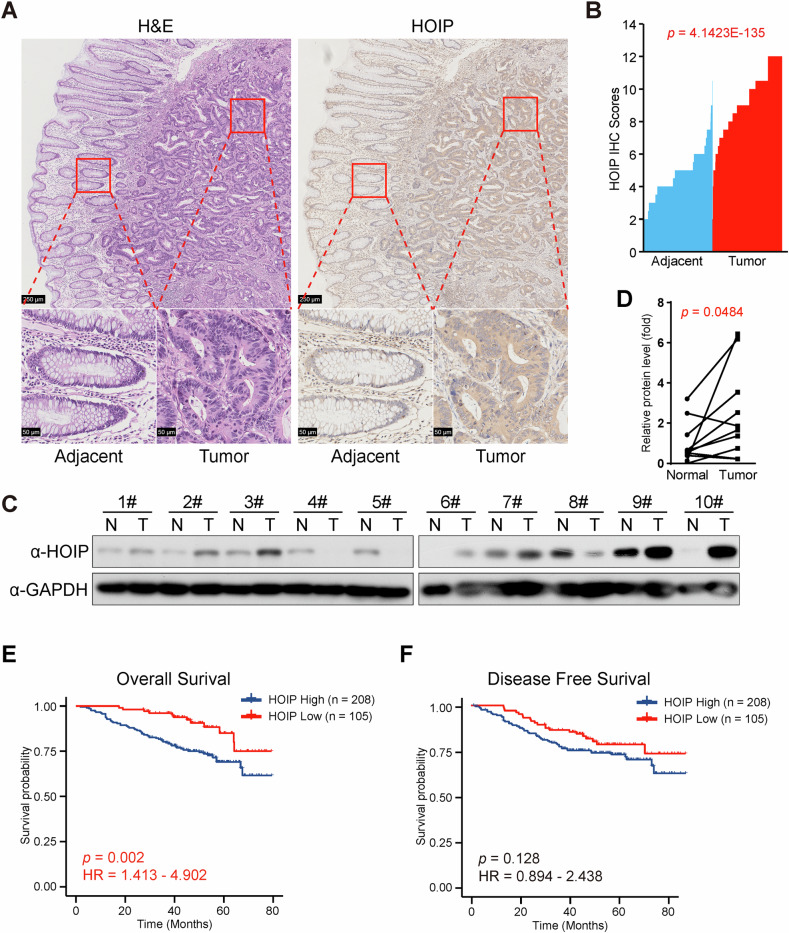
HOIP is highly expressed in CRC tissues. **A** Representative images of IHC staining of both human CRC tissues and adjacent tissues in the same section stained for HOIP. **B** HOIP expression was plotted per the IHC scores in each carcinoma and adjacent tissue. **C** Immunoblotting of 10 pairs of randomly chosen CRC samples. **D** Quantification of the protein level of HOIP in (**C**). Kaplan‒Meier estimates of the overall survival (**E**) and disease-free survival (**F**) of CRC patients between the low and high expression groups for HOIP.

As the function of linear ubiquitylation in tumor growth remains unclear, we explored the expression of each component of LUBAC in the TCGA dataset. Interestingly, all three components showed higher expression in tumor tissues in CRC (Supplementary Fig. 1A–C) and several other cancers (data not shown). For further analysis of the protein level of HOIP, the catalytic subunit in LUBAC, tumor tissues from CRC patients were collected, and the protein level of HOIP in tumor tissues and adjacent tissues was then assessed via immunohistochemistry (IHC). Consistent with its mRNA level, the protein level of HOIP was higher in tumor tissues than in adjacent tissues (Fig. 1A, B). Interestingly, HOIP expression was associated with histologic grade, TMN(T) stage and tumor general type in CRC (Table 1), and higher expression of HOIP was identified in lower differentiated tumor tissues (Supplementary Fig. 1D), which indicates the function of HOIP in CRC progression. Immunoblotting (IB) revealed that the variation in the expression level of HOIP is quite high, though in most cases (7/10), the expression of HOIP increased in tumor tissues (Fig. 1C). Additionally, the statistical analysis supports the conclusion that HOIP is highly expressed in CRC tissues (Fig. 1D).

**Table 1:** Association of HOIP levels with different clinicopathologic characteristics in CRC.

Clinicopathologic	HOIP		*p*-value
	Low	High	
Sex			
Female	56	100	0.814
Male	68	128	
Age			
<60	63	103	0.312
≥60	61	125	
Histologic grade			
G1	27	17	0.000284
G2	88	199	
G3	9	12	
Primary tumor size, cm			
<4	41	66	0.355
4‒6	55	117	
>6	27	45	
TMN stage (T)			
1	8	3	0.021
2	4	17	
3	94	168	
4	18	40	
TMN stage (N)			
0	81	152	0.952
1	29	50	
2	14	26	
TMN stage (M)			
0	114	207	0.717
1	10	21	
Dukes stage			
A-B	77	143	0.908
C-D	47	85	
Vascular invasion			
Negative	105	208	0.061
Positive	19	20	
Nerve invasion			
Negative	101	194	0.376
Positive	23	34	
General type			
Bulge type	47	56	0.031
Ulcer type	76	169	
Other type	1	3	
CEA level, ng/ml			
≤5	63	110	0.883
>5	41	81	
CA125 level, U/ml			
≤34	84	148	0.427
>34	7	22	
CA199 level, U/ml			
≤27	67	135	0.440
>27	28	39	
History of intestinal polyps			
Negative	110	204	0.945
Positive	11	18	
Chemotherapy			
Negative	56	93	0.628
Positive	62	126	

Higher protein levels of HOIP correlated with poor prognosis in CRC patients, as a shorter overall survival (OS) was discovered (Fig. 1E and Table 2), although its effect on disease-free survival (DFS) was not significant (Fig. 1F and Table 2). Analysis of the expression and prognostic data in the TCGA dataset further confirmed that the expression of HOIP, together with other components in LUBAC, was associated with poor prognosis in CRC patients (Supplementary Fig. 1E–G).

**Table 2:** Univariate and multivariate analysis of factors associated with survival and recurrence of CRC patients.

	OS								DFS							
	Univariate analysis				Multivariate analysis				Univariate analysis				Multivariate analysis			
	HR	HR 95% CI		*p*	HR	HR 95% CI		*p*	HR	HR 95% CI		*p*	HR	HR 95% CI		*p*
		Low	High			Low	High			Low	High			Low	High	
Sex	0.929	0.581	1.486	0.760					0.663	0.423	1.037	0.072				
Age	1.141	0.710	1.832	0.586					1.031	0.658	1.616	0.893				
Histologic grade	1.989	1.249	3.165	0.004	0.498	0.498	0.498	0.498	1.553	0.991	2.435	0.055				
Primary tumorsize	0.789	0.557	1.119	0.184					0.746	0.536	1.037	0.082				
TMN stage (T)	3.228	2.024	5.147	0.000	3.184	1.477	6.860	0.003	2.331	1.507	3.607	0.000	2.178	1.166	4.068	0.015
TMN stage (N)	2.531	1.903	3.367	0.000	1.667	0.816	3.405	0.161	2.111	1.590	2.803	0.000	1.611	0.817	3.178	0.169
TMN stage (M)	12.429	7.444	20.753	0.000	4.053	1.545	10.632	0.004	5.141	2.853	9.264	0.000	1.448	0.573	3.659	0.433
Dukes stage	5.628	3.348	9.462	0.000	1.040	0.322	3.358	0.948	3.622	2.289	5.732	0.000	0.837	0.277	2.530	0.753
Vascular invasion	2.955	1.667	5.239	0.000	1.448	0.598	3.509	0.412	2.185	1.203	3.969	0.010	1.334	0.610	2.917	0.471
Nerve invasion	2.580	1.527	4.359	0.000	1.068	0.457	2.497	0.879	1.346	0.753	2.407	0.316				
General type	2.173	1.257	3.758	0.005	1.427	0.552	3.691	0.463	1.890	1.131	3.159	0.015	1.584	0.722	3.479	0.251
CEA level	2.606	1.489	4.561	0.001	1.380	0.648	2.939	0.404	1.987	1.195	3.303	0.008	1.122	0.599	2.102	0.719
CA125 level	2.066	1.029	4.149	0.041	0.858	0.344	2.140	0.742	1.550	0.760	3.164	0.228				
CA199 level	3.498	1.994	6.137	0.000	1.684	0.752	3.770	0.205	3.349	1.982	5.659	0.000	2.054	1.085	3.888	0.027
History of intestinal polyps	0.741	0.269	2.037	0.561					0.638	0.233	1.749	0.383				
Chemotherapy	3.129	1.698	5.768	0.000	1.725	0.748	3.976	0.201	3.532	1.964	6.353	0.000	2.851	1.344	6.048	0.006
HOIP expression	2.632	1.413	4.902	0.002	2.604	1.030	6.581	0.043	1.476	0.894	2.438	0.128				

In summary, our findings revealed the oncogenic role of LUBAC in CRC, and the expression of HOIP was associated with the differentiation grade of tumor tissues and the overall survival of CRC patients.

### HOIP facilitates CRC cell growth

Further experiments are required to illustrate whether LUBAC facilitates tumor growth and progression, although clinical analysis revealed the relationship between LUBAC and CRC.

**Fig. 2:**
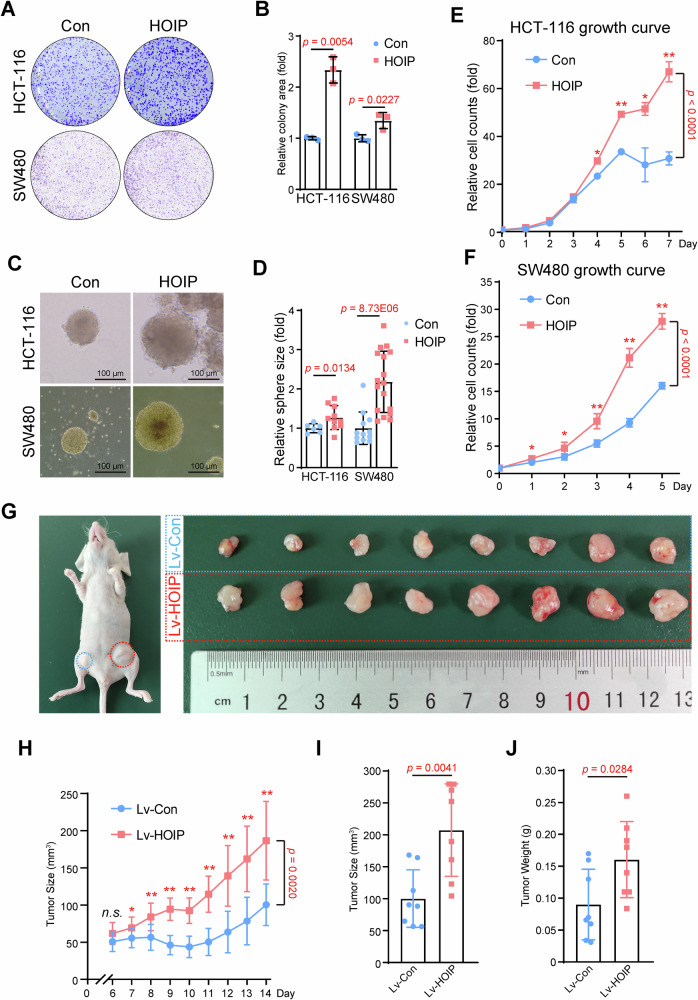
HOIP facilitates CRC cell growth. **A** HOIP promotes the colony formation of CRC cells. HCT-116 or SW480 cells stably expressing LV-HOIP were seeded in a 6-well plate for 14 days. **B** Quantitative analysis of HCT-116 and SW480 cells stably overexpressing HOIP. Data are shown as the means ± SDs, *n* = 3. **C** HOIP promotes the sphere formation of CRC cells. HCT-116 or SW480 cells stably expressing LV-HOIP were mixed with soft agar (0.3%) and seeded in a 12-well plate for 14 days. **D** Quantitative analysis of HCT-116 and SW480 cells stably overexpressing HOIP. Data are shown as the means ± SDs. Growth curve of CRC cells. HCT-116 (**E**) or SW480 (**F**) cells stably expressing LV-HOIP were seeded in a 96-well plate, and the cells were counted by Cell Counting Kit (CCK)-8. Data are shown as the means ± SDs, *n* = 5. **G**–**J** HOIP overexpression promotes tumor growth ex vivo. HCT-116 stable cell lines (1 × 10^7^ cells) that overexpressed HOIP were subcutaneously injected into eight nude mice on each side of the inguinal region. Xenografts were harvested after 2 weeks. Tumor sizes on either side were monitored every other day (**H**). Tumor size (**I**) and tumor weight (**J**) were determined. Data are shown as the means ± SDs, *n* = 8.

Ectopic expression of HOIP was constructed in HCT-116 and SW480, two different CRC cell lines (Fig. S2A). Colony formation assays revealed that HOIP promotes tumor cell growth in both CRC cell lines (Fig. 2A, B), and this conclusion was further proven in sphere formation assays in soft agar (Fig. 2C, D). Moreover, cell viability was tested via a CCK-8 kit every day to generate cell growth curves, and the results showed that HOIP increased cell proliferation in CRC cells (Fig. 2E, F).

To further test the effect of HOIP in CRC, we generated a xenograft model in nude mice (Fig. 2G). The results showed that ectopic expression of HOIP led to faster growth of CRC tumors (Fig. 2H), and both tumor size (Fig. 2G, I) and tumor weight (Fig. 2J) were significantly increased. IHC staining revealed that HOIP expression increased cell proliferation, as the number of Ki67-positive cells was increased (Supplementary Fig. 2B).

In summary, this study illustrated the association between HOIP and CRC and further demonstrated that HOIP facilitates tumor growth in CRC cells both in vitro and ex vivo.

### HOIP inhibition suppresses CRC cell growth

In contrast, the effect of HOIP depletion was also assessed in HT-29 and HCT-116 cells with stable HOIP interference by shRNAs (Supplementary Fig. 3A). In both cell lines, knockdown of HOIP decreased colony formation (Supplementary Fig. 3B, C) and depressed sphere formation (Supplementary Fig. 3D, E), indicating the inhibitory effect of HOIP depletion on proliferation in CRC cells.

**Fig. 3:**
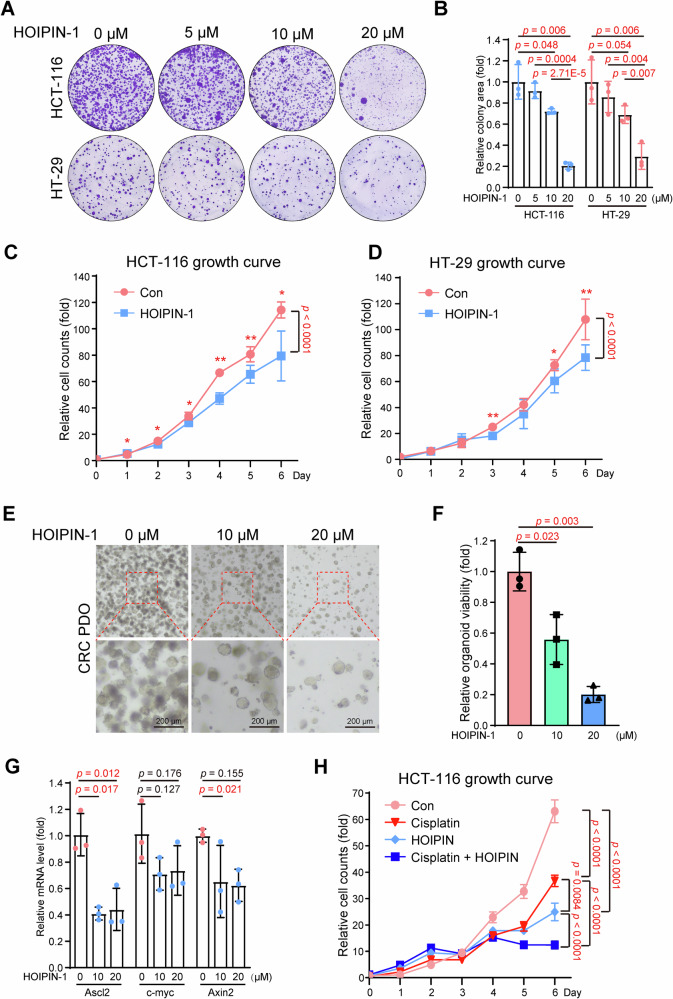
HOIP inhibition suppresses CRC cell growth. **A** HOIPIN-1 inhibited the colony formation of CRC cells. HCT-116 or HT-29 cells were seeded in a 6-well plate and then treated with HOIPIN-1 at the indicated concentration for 14 days. **B** Quantitative analysis of HCT-116 and HT-29 cells treated with HOIPIN-1. Data are shown as the means ± SDs, *n* = 3. Growth curve of CRC cells. HCT-116 (**C**) or HT-29 (**D**) cells were seeded in a 96-well plate and then treated with or without HOIPIN-1 (5 μM). The cells were counted by CCK-8. Data are shown as the means ± SDs, *n* = 3. **E** HOIPIN-1 inhibited the colony formation of PDOs derived from CRC. PDOs treated with HOIPIN-1 at the indicated concentration were cultured for 11 days, and images were taken at the end point. **F** Viability of PDOs treated with HOIPIN-1 were measured by CCK-8. Data are shown as the means ± SDs, *n* = 3. **G** Expression of indicated genes in PDOs were determined by qPCR. Data are shown as the means ± SDs, *n* = 3. **H** HOIPIN-1 cooperates with cisplatin in suppressing cell growth. HCT-116 cells were seeded in a 96-well plate and then treated with HOIPIN-1 (10 μM) and cisplatin (1 μM) or not. The cells were counted by CCK-8. Data are shown as the means ± SDs, *n* = 5.

Masahiro Tamaru and his collaborators identified and synthesized several small molecules targeting LUBAC, which were previously named HOIPINs [26, 27], and Fuminori Tokunaga et al. further confirmed that HOIPIN-1 and HOIPIN-8 could block linear ubiquitylation by bonding to HOIP and suppressing LUBAC by functional assays and crystal structures [28]. Therefore, we tested the effect of LUBAC inhibition via small molecules in CRC cells. Colony formation assays revealed that HOIPIN-1 suppressed tumor cell growth in HCT-116 and HT-29 cells in a dose-dependent manner (Fig. 3A, B), and cell growth curves also showed a similar conclusion (Fig. 3C, D).

To further explore the potential of HOIPIN-1 in CRC therapy, we used patient-derived organoids (PDOs) from CRC tissue. As shown in Fig. 3E and F, HOIPIN-1 treatment decreased the number of PDOs, and the expression of *Ascl2* and *Axin2* was suppressed (Fig. 3G), indicating the suppressive effect of HOIPIN-1 on stemness and cell proliferation in CRC organoids. Interestingly, HOIPIN-1 suppressed the growth of organoids derived from CRC but not those derived from normal intestinal (Supplementary Fig. 3F, G), which indicates a content-dependent function of LUBAC in tumor or normal tissues.

As platinum drugs are widely used for clinical chemotherapy in CRC, we investigated the effect of combined treatment with cisplatin and HOIPIN-1 in CRC cells. As shown in Fig. 3H, either HOIPIN-1 or cisplatin suppressed cell growth in HCT-116 cells, and the combination of these two molecules led to a dramatic inhibitory effect on CRC cells, indicating that the combination of HOIPIN-1 and other chemotherapy drugs is a promising strategy in CRC therapy.

Conclusively, inhibition of LUBAC through RNA interference or small molecules led to significant suppression of cell growth in both CRC cells and PDOs, indicating that LUBAC is a novel and promising therapeutic target in CRC.

### HOIP interacts with Gli proteins

Potential linear polyubiquitylation substrates were screened previously [4], which revealed that Gli proteins were promising targets of linear ubiquitination. Therefore, the interaction between Gli proteins and LUBAC was examined.

**Fig. 4:**
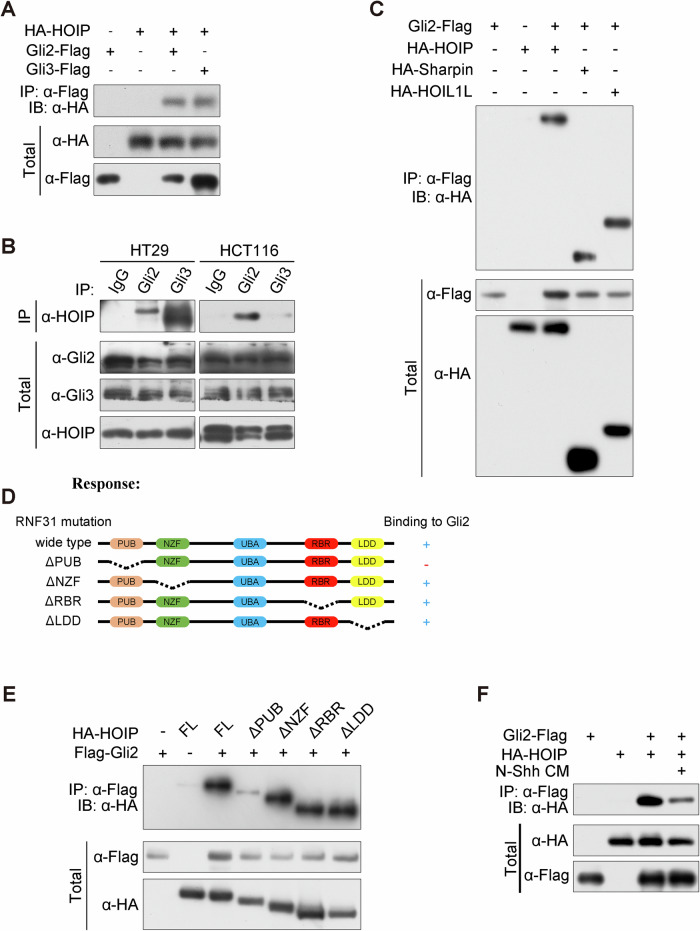
HOIP interacts with Gli proteins. **A** Ectopically expressed HOIP interacts with Gli proteins. HEK-293T cells transfected with HA-HOIP and Flag-Gli2 or Flag-Gli3 plasmids were subjected to a co-IP assay. **B** Endogenous complexes of HOIP with Gli proteins. Cell lysates of HCT-116 or HT-29 cells were subjected to IP analysis with the indicated antibodies and protein A/G beads overnight. Bound proteins were analysed via IB. **C** Gli2 interacts with LUBAC components. HEK-293T cells transfected with HA-HOIP, Sharping or HOIL and Flag-Gli2 plasmids were subjected to a co-IP assay. **D** Schema of domains of HOIP. **E** HOIP binds to Gli2 through its PUB domain. HEK-293T cells transfected with Flag-Gli2 and HA-HOIP or its truncated mutation plasmids were subjected to a co-IP assay. **F** Hh signaling affects the interaction between HOIP and Gli2. HEK-293T cells transfected with Flag-Gli2 and HA-HOIP plasmids, followed by treatment with N-Shh for 30 min, were subjected to a co-IP assay.

Immunoprecipitation revealed that both Gli2 and Gli3 could bind to HOIP exogenously and endogenously (Fig. 4A, B), and all three subunits in LUBAC interact with Gli2 and Gli3 (Fig. 4C and Supplementary Fig. 4A). To map the interactive domain that mediates binding to Gli proteins, we generated a set of truncated mutations (Fig. 4D). Further immunoprecipitation illustrated that HOIP interacts with Gli2 through its PUB domain (Fig. 4E and Supplementary Fig. 4B). Furthermore, as the phosphorylation and degradation of Gli proteins are regulated by upstream Hh signaling, we investigated whether Hh signaling affects the interaction between LUBAC and Gli proteins. As shown in Fig. 4F, treatment with N-Shh, the activated ligand of Hh signaling, partially suppressed the interaction between Gli2 and HOIP.

In conclusion, Gli proteins could bind to LUBAC, and the PUB domain of HOIP mediates this interaction.

### HOIP stabilizes Gli proteins and activates Hh signaling

**Fig. 5:**
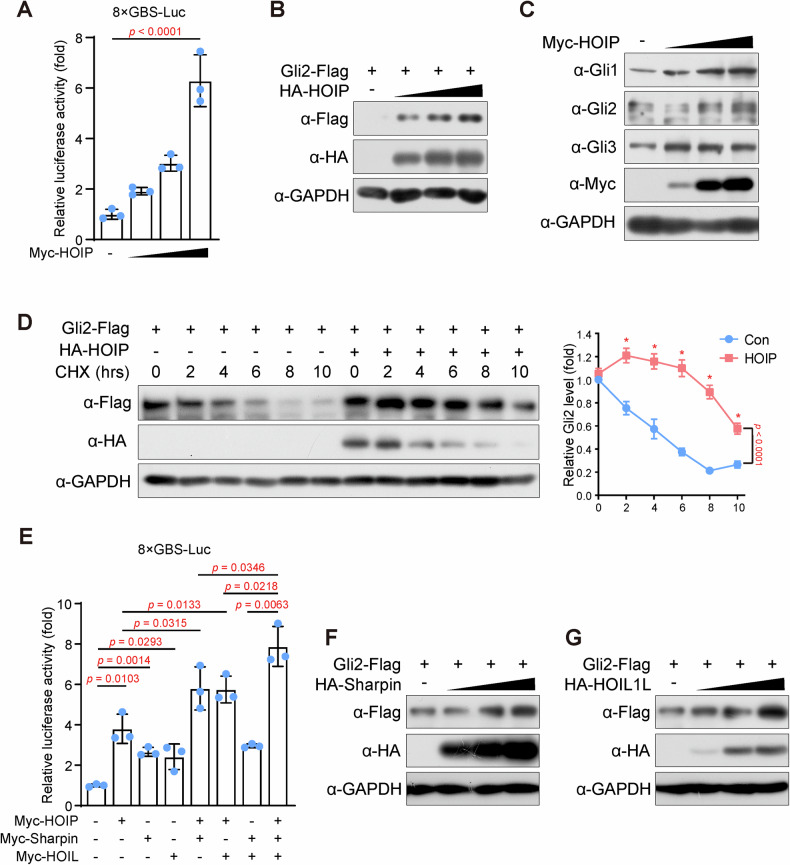
HOIP mediates the linear polyubiquitylation of Gli proteins. **A** HOIP activates Hh signaling. HCT-116 cells were harvest after transfected with 8×GBS-Luc, pRL-TK, and HA-HOIP for 48 hours, and the cell lysate were subjected to a dual-luciferase reporter assay according to the manual. The firefly luciferase activity was normalized to Renila luciferase activity. Data are shown as the means ± SDs, *n* = 3, and ANOVA with multiple comparisons was investigated. **B** HOIP elevates the protein level of exogenous Gli2. HEK-293T cells transfected with Flag-Gli2 and HA-HOIP (0, 0.15 μg, 0.3 μg and 0.6 μg per well in 12 well-plate) plasmids were subjected to IB with the indicated antibodies. **C** HOIP elevates the protein level of endogenous Gli2 and Gli3. HEK-293T cells transfected with HA-HOIP (0, 0.15 μg, 0.3 μg and 0.6 μg per well in 12 well-plate) plasmids were subjected to IB with the indicated antibodies. **D** HOIP impairs the degradation of Gli2. Cycloheximide (CHX) (100 μg/ml) was incubated for the indicated period with HEK-293T cells transfected with or without Flag-Gli2 and HA-HOIP. Cell lysates were harvested for IB with the indicated antibody. **E** LUBAC activates Hh signaling. HCT-116 cells transfected with 8×GBS-Luc, pRL-TK, HA-HOIP, Sharpin and HOIL were subjected to a dual-luciferase reporter assay. Data are shown as the means ± SDs, *n* = 3. **F**, **G** LUBAC stabilizes Gli2. HEK-293T cells transfected with Flag-Gli2 and HA-Sharpin or HOIL (0, 0.15 μg, 0.3 μg and 0.6 μg per well in 12 well-plate) plasmids were subjected to IB with the indicated antibodies.

As Gli proteins are the core transcription factors in Hh signaling, we examined the activity of Hh signaling via dual-luciferase reporter assays. The luciferase assay based on 8xGBS-luciferase specifically response to the activation of Hh signaling, as it contains 8 Gli binding sites (GBS) in its promoter region, and Gli proteins, activated by Hh signaling, would binds to the reporter region and facilitate the transcription of luciferase. The results showed that ectopic expression of HOIP elevated 8xGBS reporter activity in a dose-dependent manner (Fig. 5A), indicating that HOIP activates Hh signaling. As the accumulation of full-length Gli proteins is a marker of Hh signaling activity, the effect of LUBAC on the levels of Gli proteins was examined. Interestingly, HOIP elevated the protein levels of all three Gli proteins (Fig. 5B and Supplementary Fig. 5A, B) in a dose-dependent manner, and endogenous Gli proteins were also increased in cells with HOIP expression (Fig. 5C), indicating that HOIP might stabilize Gli proteins. Moreover, degradation of Gli proteins was assessed by blocking protein synthesis with cycloheximide (CHX), and the results showed that the half-life of both Gli2 and Gli3 was dramatically extended in HOIP-expressing cells (Fig. 5D and Supplementary Fig. 5C), directly determining the regulatory function of HOIP on the stability of Gli proteins. These experiments illustrated that HOIP could activate Hh signaling by stabilizing Gli proteins.

Moreover, Sharpin and HOIL activate Hh signaling individually, and cooperation among these subunits further activates Hh signaling (Fig. 5E), indicating that activation of Hh signaling by HOIP or other subunits requires whole LUBAC activity. Furthermore, the effects of Sharpin and HOIL were examined, and similar results were obtained: both Sharpin and HOIL increased the protein levels of Gli2 and Gli3 (Fig. 5F, G and Supplementary Fig. 5D, E), indicating that LUBAC regulates the stability of Gli proteins. Interestingly, HOIP also showed a more effective role in stabilizing Gli2 and activating Hh signaling (Fig. 5E–G), and as shown in Supplementary Fig. 1A–C, the catalytic subunit HOIP had the lowest expression level, suggesting that the level of HOIP is more critical in regulating the activity of LUBAC.

In summary, LUBAC could activate Hh signaling by stabilizing Gli proteins.

### HOIP mediates the linear polyubiquitylation of Gli proteins

**Fig. 6:**
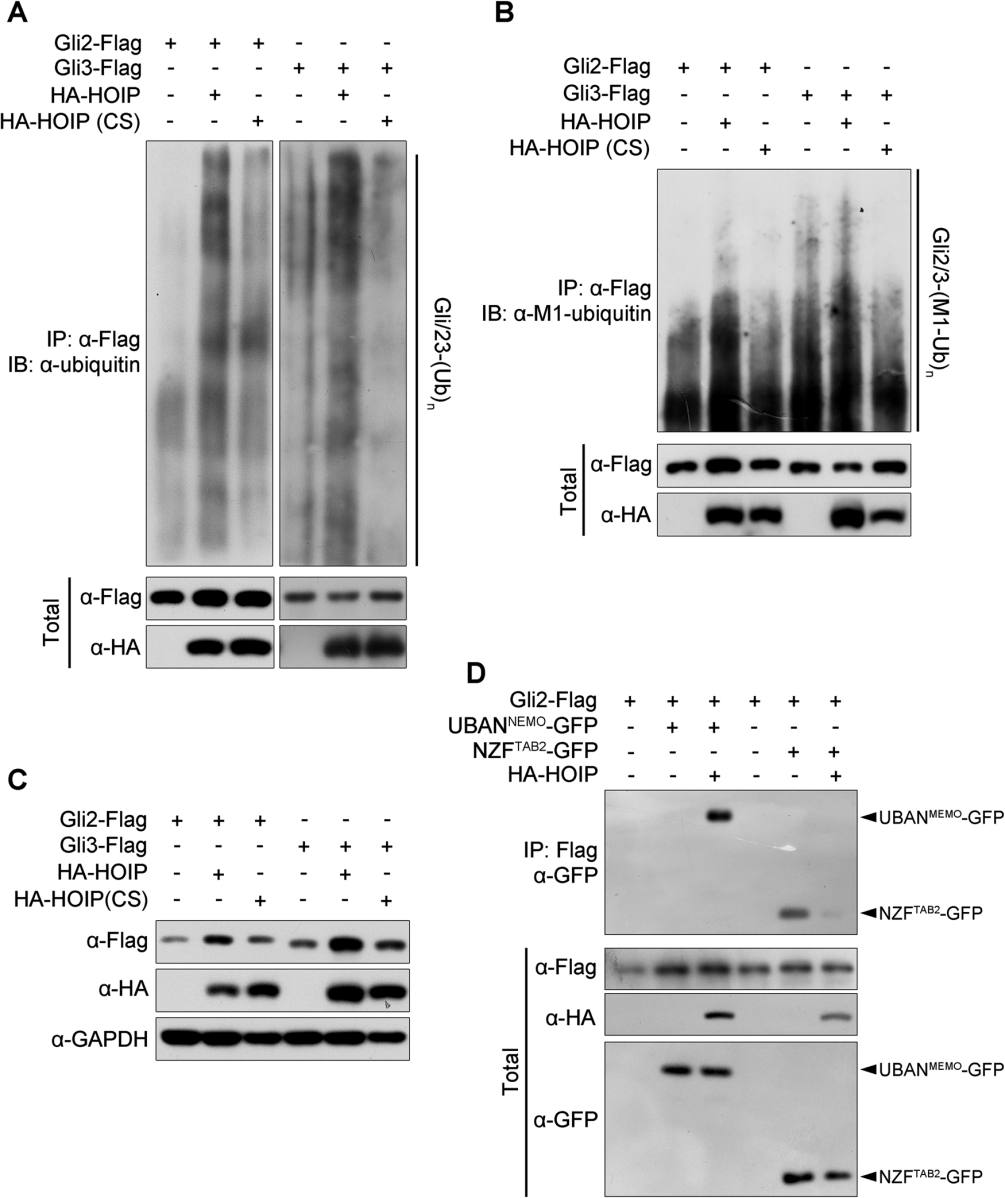
HOIP mediates the linear polyubiquitylation of Gli proteins. **A** HOIP mediates the polyubiquitylation of Gli proteins. HEK-293T cells transfected with Flag-Gli2 or Gli3 and HA-HOIP or its point mutation plasmids were subjected to a denatured co-IP assay. **B** HOIP mediates the linear ubiquitylation of Gli proteins. HEK-293T cells transfected with Flag-Gli2 or Gli3 and HA-HOIP or its point mutation plasmids were subjected to a denatured co-IP assay. Antibodies specifically recognizing M1-polyubiquitylation were used to identify linear ubiquitin chains. **C** E3 ligase activity of HOIP is essential for the stabilization of Gli proteins. HEK-293T cells transfected with Flag-Gli2 or Gli3 and HA-HOIP or its point mutation plasmids were subjected to IB with the indicated antibodies. **D** HOIP mediates linear ubiquitylation but impairs the K63 ubiquitination of Gli proteins. HEK-293T cells transfected with Flag-Gli2 and HA-HOIP or its point mutant, together with GFP-UBAN^NEMO^ or GFP-NZF^TAB2^ plasmids, were subjected to a denatured co-IP assay. UBAN^NEMO^ was used to recognize M1 ubiquitination, and NZF^TAB2^ was used to specifically recognize K63 ubiquitination.

As ubiquitin E3 ligases, it is easy to guess that HOIP and LUBAC may mediate the ubiquitylation of Gli proteins. Indeed, HOIP mediates the polyubiquitylation of Gli2 and Gli3, whereas mutations (C699, 702, 871, 874S, CS for short) in its RBR domain destroying its E3 ligase activity blocked the ubiquitination of Gli2 and Gli3 mediated by HOIP (Fig. 6A). Furthermore, an antibody against the linear ubiquitin chain specifically was investigated in this ubiquitination assay, and the results further confirmed that the ubiquitin chain conjugated to Gli2 and Gli3 mediated by HOIP was linear (Fig. 6B). Consistently, the CS mutation lost the ability to elevate the protein levels of Gli2 and Gli3 (Fig. 6C) and the capability to stabilize Gli3 (Supplementary Fig. 6A).

In addition, some ubiquitin-binding domains can recognize different types of ubiquitin chains; for example, the UBAN domain of NEMO (UBAN^NEMO^) interacts with linear ubiquitin chains with a relatively high affinity [29], and the NZF (NZF^TAB2^) domain of TAB2 binds to K63-linked ubiquitin chains [30, 31], which could be utilized as a sensor to monitor these ubiquitin chains [32]. The results showed that HOIP elevated the linear ubiquitylation of Gli2, as its interaction with UBAN^NEMO^ was dramatically increased, whereas the K63-linked ubiquitination of Gli2 was suppressed, as its binding to NZF^TAB2^ decreased (Fig. 6D), which further concluded that HOIP mediates the linear ubiquitylation of Gli proteins, but not the branched ubiquitylation.

In summary, our findings showed that HOIP mediates the linear ubiquitylation of Gli proteins, and this modification is essential for its protein stability.

## Discussion

**Fig. 7:**
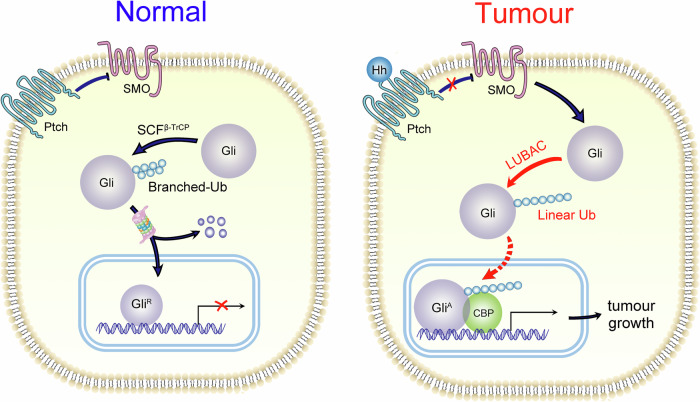
Linear ubiquitylation of Gli proteins facilitates CRC growth. LUBAC mediates the linear ubiquitylation of Gli proteins, which regulates their protein stability and activates Hh signalling, therefore facilitates cell growth in CRC.

In this study, we identified Gli proteins as novel substrates of linear ubiquitylation mediated by HOIP, which was further shown to stabilize Gli proteins and to activate Hh signaling (Fig. 7). The function of HOIP in CRC was also determined. HOIP facilitates tumor growth in CRC cells, both ex vivo and in vitro. The expression of HOIP was relatively higher in tumor tissues than in adjacent tissues in CRC patients, and higher HOIP expression was related to poor prognosis, further indicating its function in promoting tumor progression. As expected, inhibition of LUBAC via RNA interference or small molecules suppressed cell growth in both CRC cells and PDOs, which indicates that LUBAC is a promising target in CRC therapy. Furthermore, the combination of a LUBAC inhibitor and cisplatin led to a dramatic suppression of cell growth in CRC cells, further determining the potential value of LUBAC inhibition in clinical CRC therapy. Therefore, our findings identified LUBAC as a novel therapeutic target, and the use of small molecules targeting LUBAC, such as HOIPIN-1, might be a promising therapeutic strategy in CRC.

The functions of LUBAC in tumorigenesis and progression remain unclear, although its function in immunity [2] and angiogenesis [9, 10] has been fully identified. Recently, it has been reported that linear ubiquitination of PTEN impairs its function and promotes tumor progression in prostate cancer [15]. Due to the limited understanding of LUBAC in cancer, though small molecules targeting LUBAC have been proposed to benefit immune disorders, less focus has been placed on its potential usage in cancer therapy. Herein, we offer novel insight into these antagonists of LUBAC and linear polyubiquitylation, which is promising in tumor therapy.

A recent study determined the content-dependent function of LUBAC in mouse intestinal epithelia. Loss of LUBAC in the epithelial cells normally had no suppressive function; however, Lipopolysaccharide (LPS) treatment led to apoptosis and cell death only in intestinal epithelia with LUBAC deficiency but not the wild type epithelia [33]. Interestingly, we also observed the content-dependent mechanism of LUBAC inhibition between normal and tumor epithelia. In contrast to that of organoids derived from CRC tissue, the growth of organoids derived from normal intestinal was resistant to HOIPIN-1, although the detailed mechanism remains unclear. The function of LUBAC in cell proliferation differs in normal intestinal epithelial cells and tumor cells, which makes this antagonist more suitable for potential clinical usage.

Intestinal epithelial cells secrete Hh ligands to activate Hh signaling and facilitate proliferation in stromal cells; however, Hh signaling is not activated and does not regulate cell growth directly in intestinal epithelial cells [34]. Interestingly, higher levels of Gli proteins, representing the aberrant activation of Hh signaling, can be observed in CRC, which promotes tumor growth and progression [35, 36]. The different states of Hh signaling in normal epithelia and tumors make it a potential therapeutic target; however, an inhibitor of SMO, a key regulator of Hh signaling upstream of Gli proteins, failed in a phase II trial [37]. Herein, we reported that the accumulation of Gli proteins in CRC is probably due to the dysregulation of its ubiquitylation and degradation, independent of upstream signaling. In addition, the inactivation of Hh signaling in normal intestinal epithelia could partially explained why HOIP inhibition had no suppressive effect in intestinal organoids derived from normal intestine.

Interestingly, the interaction between HOIP and Gli proteins was attenuated by Shh treatment. Given that HOIP can stabilize Gli proteins and activate Hh signaling, the suppressive effect of Shh in disrupting HOIP-Gli binding might contribute to a negative feedback loop, limiting the accumulation of Gli proteins and the activation of Hh signaling. However, more evidence is required for this hypothesis.

Ubiquitylation is essential in regulation of protein stability and other biological processes. In most cases, polyubiquitylation marks its substrates for degradation, as K11-, K29-, K48-, and K63-linked ubiquitin chains might participate in protein degradation via the proteasome or lysosome, although K11- and K48-linked branches trigger degradation more frequently than other types [38]. The knowledge of linear ubiquitylation, however, remains limited. Recent reports found that linear polyubiquitylation of ATG13 [7] and GPx4 [39] mediated by HOIP protects them from proteasomal degradation, indicating that this atypical ubiquitylation also participates in regulation of protein stability. Similarly, our findings also revealed the stability regulatory function of linear ubiquitylation, which stabilizes Gli proteins in our case. The detailed mechanism how the linear ubiquitin chain protects its substrates from proteasomal degradation remains unclear. Recently, linear ubiquitylation was reported to release CP110 from CEP97 [8], which indicates that linear ubiquitylation might impair protein-protein interactions. Linear ubiquitylation of Gli proteins probably attenuates its interaction with other E3 ligases, but more evidence is required.

HOIP, HOIL-1, and SHARPIN were reported to exist largely in stable LUBAC complexes [40], and three components share similar abundance in cells [41] suggesting that the levels of LUBAC subunits are closely coordinated [42]. However, multiple reports have declared that all three key components of LUBAC can mediate different types of ubiquitylation independent of LUBAC. For example, monoubiquitylation of p53 mediated by HOIP, which expedites its degradation, is well understood in breast cancer and CRC [12, 43]. How the ubiquitylation type of its substrates is regulated remains an open question. Recent reports found that PTEN, a novel substrate of linear polyubiquitylation, interacts with HOIP through its NZF domain [15], and our findings revealed that Gli2 binds to HOIP via its PUB domain, which indicates that the interacting domain on HOIP is not essential to determine the type of ubiquitin chain on its substrates. Interestingly, PTEN and GPx4, two identified substrates of linear polyubiquitylation, were also reported to interact with Sharpin [15, 39], another subunit in LUBAC, and Gli proteins were also found to bind to Sharpin. Therefore, it seems that the proper interacting couples, including both HOIP and Sharpin, might be crucial for the ubiquitin-chain type.

In summary, our findings identified Gli proteins as novel substrates of linear polyubiquitylation, which facilitates Hh signaling and promotes cell proliferation in CRC.

## Subjects and methods

### Patients and clinical specimens

A cohort containing 314 patients diagnosed with primary CRC who underwent surgical resection at the First Affiliated Hospital of Nanchang University was included in this study. All patients were diagnosed via histopathological criteria and had not received chemotherapy or immunotherapy before surgery. The tissue samples were reviewed by a pathologist to ensure that they contained both tumor and adjacent non-malignant tissues, and the normal tissue was defined as the tissue located more than 1 cm away from the tumor. Detailed clinical and pathological information is summarized in Table 1.

### Immunoblotting (IB) and immunohistochemistry (IHC)

Total protein extracts were harvested and subjected to immunoblotting as described previously [44] with the indicated primary antibodies (Supplementary Table 1). The immunoblot films were digitalized with an Epson V700 scanner, and the intensity of major bands was quantitated using ImageJ (National Institutes of Health, Bethesda, MD, USA). Each experiment was repeated at least three times. The excised clinical or animal tissues were fixed in a 10% neutral buffered formalin solution, dehydrated and embedded into paraffin wax blocks. Embedded tissues were cut into 3-μm sections, mounted onto slides, and processed for histopathological evaluation. All samples were stained with haematoxylin and eosin (H&E), and IHC procedures were performed as described previously [21].

### Cell lines and transfection

HCT-116, SW480 and HEK-293T cells were purchased from the Cell Bank of the Type Culture Collection of the Chinese Academy of Sciences (Shanghai, China) in 2018, and HT-29 cells were purchased from the American Type Culture Collection (ATCC, Manassas, VA, USA) in 2016. HEK-293T and SW480 cells were cultured in Dulbecco’s modified Eagle’s medium (DMEM) (C11995500BT; Gibco; Grand Island; NY; USA), and HCT-116 and HT-29 cells were cultured in McCoy’s 5A (Modified) Medium (16600082; Gibco; Grand Island; NY; USA), all with 10% FBS (10091148; Gibco) at 37 °C in a humidified 5% CO_2_ atmosphere. CRC cells were further authenticated by Short tandem repeat (STR) analysis, and PCR were investigated to check the mycoplasma infection of the cells. Cells were transiently transfected with Lipofectamine 2000 for HCT-116 and HEK-293T cells according to the manufacturer’s instructions. In all experiments, the medium was replaced daily.

### Plasmid construction and lentivirus

cDNAs of HOIP, Sharpin, HOIL, TAB2 and NEMO were kind gifts from Dr. Jiahuai Han, Xiamen University, and the coding sequences were amplified by PCR, cut with the indicated restriction enzymes, and inserted into pcDNA3.1 or pEGFP-N3 to construct plasmids expressing the indicated genes or segments. Plasmid of Gli3 was a gift from Martin Fernandez-Zapico (Addgene plasmid # 84921), and other plasmids were preserved in our laboratory previously. Point mutations of HOIP were generated with a site-directed mutagenesis kit (Code No. SMK-101, TOYOBO, Japan) following the manufacturer’s instructions. Short hairpin RNAs (shRNAs) targeting HOIP were synthesized by Sangon Biotech (Shanghai) Co., Ltd., and inserted into pSuper. Lentivirus expressing HOIP was purchased from Fulengen Co., Ltd. (Guangzhou, China), and lentivirus expressing shRNAs targeting HOIP was packaged in our laboratory.

### Cell proliferation assays

For calculation of the cell growth curve, CRC cells with stable HOIP expression or knockdown were seeded in 96-well plates at a density of 1,000 cells per well in triplicate, and cell viability was measured via Cell Counting Kit-8 (CCK8, Beyotime Biotechnology, Shanghai, China) every 24 h for 5-7 consecutive days after plating. The final data are displayed as the fold increase relative to the cell viability on Day 1. The clonogenic assay procedures were described previously [45]. CRC cells (2000 cells per well) were seeded in 6-well plates and cultured for approximately 2 weeks, and the culture medium was changed every 3 days. The colonies were fixed with ethanol and stained with 0.5% crystal violet. Then, the plates were washed with phosphate-buffered saline (PBS) 3 times and left to dry at room temperature. The Epson V700 scanner was used to scan and acquire a clear image, which was quantified using ImageJ software. The soft agar colony formation assay was performed as described previously [19], and CRC cells (2000 cells per well) were seeded in 12-well plates in growth medium containing 0.35% agar (0.5 ml per well) on top of a layer of growth medium containing 0.6% agar (0.75 ml per well). Growth medium (0.5 ml) was added to the top of the agar, which was replenished every three days. The cells were cultured for approximately 2 weeks. One representative field for each filter is shown in the figures.

### Patient-delivered organoids

CRC patient tumor tissues were washed with cold HBSS. After removal of the muscle tissue, the epithelial tumor tissues were cut into small pieces and centrifuged to collect the tumor fraction. These tumor fractions were embedded in Matrigel (BD Biosciences, 356231) and seeded on 24-well plates. After Matrigel polymerization, culture medium, which included Advanced DMEM/F12 with penicillin/streptomycin, GlutaMAX, N2, B27, N-acetylcysteine, nicotinamide (10 mM, Sigma-Aldrich), and EGF (50 ng/mL, Novoprotein), was added and refreshed every 2 days. HOIPIN-1 was bought from MedChem Express (US, cat. No. HY-122881).

### Subcutaneous xenograft assay

For in vivo experiments, 1 × 10^7^ HCT-116 cells stably expressing HOIP or not were digested by trypsin, resuspended in 200 μL of sterile PBS and then injected subcutaneously into the flanks of 4-week-old female BALB/c-nu athymic nude mice (SLAC Laboratory Animal Co., Ltd., Shanghai, China). Subcutaneous tumor formation was observed from 6 days post-injection. Tumor sizes were measured every 2 days using Vernier callipers. Tumor volume was calculated with the following formula: tumor volume = (length × width^2^)/2. At 14 days after injection, tumors were harvested for immunohistochemistry and western blot analysis. Protocols for animal experiments were approved by the Ethical Committee of the First Affiliated Hospital of Nanchang University and conformed to the guidelines of the National Institutes of Health on the Ethical Use of Animals. All surgeries were performed under sodium pentobarbital anaesthesia, with minimized suffering.

### RNA extraction and q-PCR

Total RNA from organoids was extracted with TRIzol Reagent (Thermo Fisher Scientific, 15596026). RNA was reverse-transcribed into cDNA using ReverTra Ace-α kit (TOYOBO, FSK-101). q-PCR was carried out with NovoStart SYBR qPCR SuperMix Plus (Novoprotein, E096-01A) on LightCycle 480II PCR system (Roche). All primer sequences are shown in Supplementary Table 2 and purchased from Sangon Biotech.

### Statistical analysis

Differences in quantitative data between two groups were analysed using two-sided paired or unpaired Student’s t tests. The χ² test was used to analyse the correlation between gene expression and clinicopathological characteristics. The Kaplan‒Meier method and the log-rank test were performed for survival analysis. The Cox proportional hazards model was used to determine independent factors influencing survival and recurrence based on the variables selected from the univariate analysis.

### Supplementary information

Supplementary Figure 1

Supplementary Figure 2

Supplementary Figure 3

Supplementary Figure 4

Supplementary Figure 5

Supplementary Figure 6

Supplementary Figure Legends

Supplementary Table 1

Supplementary Table 2

Full-sized immunoblotting films

## Data Availability

All data of this manuscript are included in the main text and supplementary files.
