# Supplementary figures and images for: Linear polyubiquitylation of Gli protein regulates its protein stability and facilitates tumor growth in colorectal cancer
Source: Cell Death Discov. 2024 Aug 20;10:369. doi: 10.1038/s41420-024-02147-4 (PMC11335874; doi:10.1038/s41420-024-02147-4)

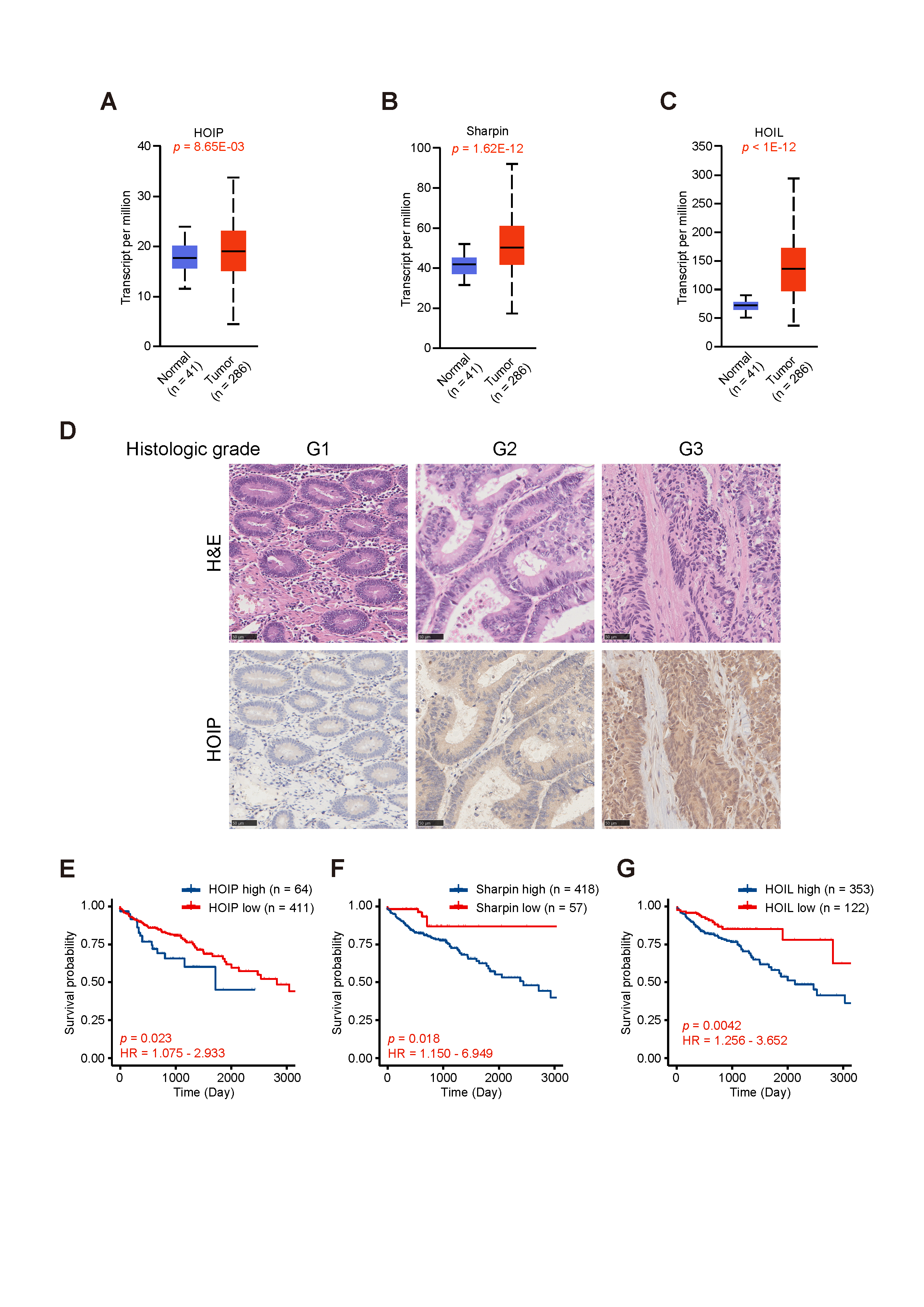

Supplement: Supplementary file 1 — Supplementary Figure 1 [file 41420_2024_2147_MOESM1_ESM.tif]

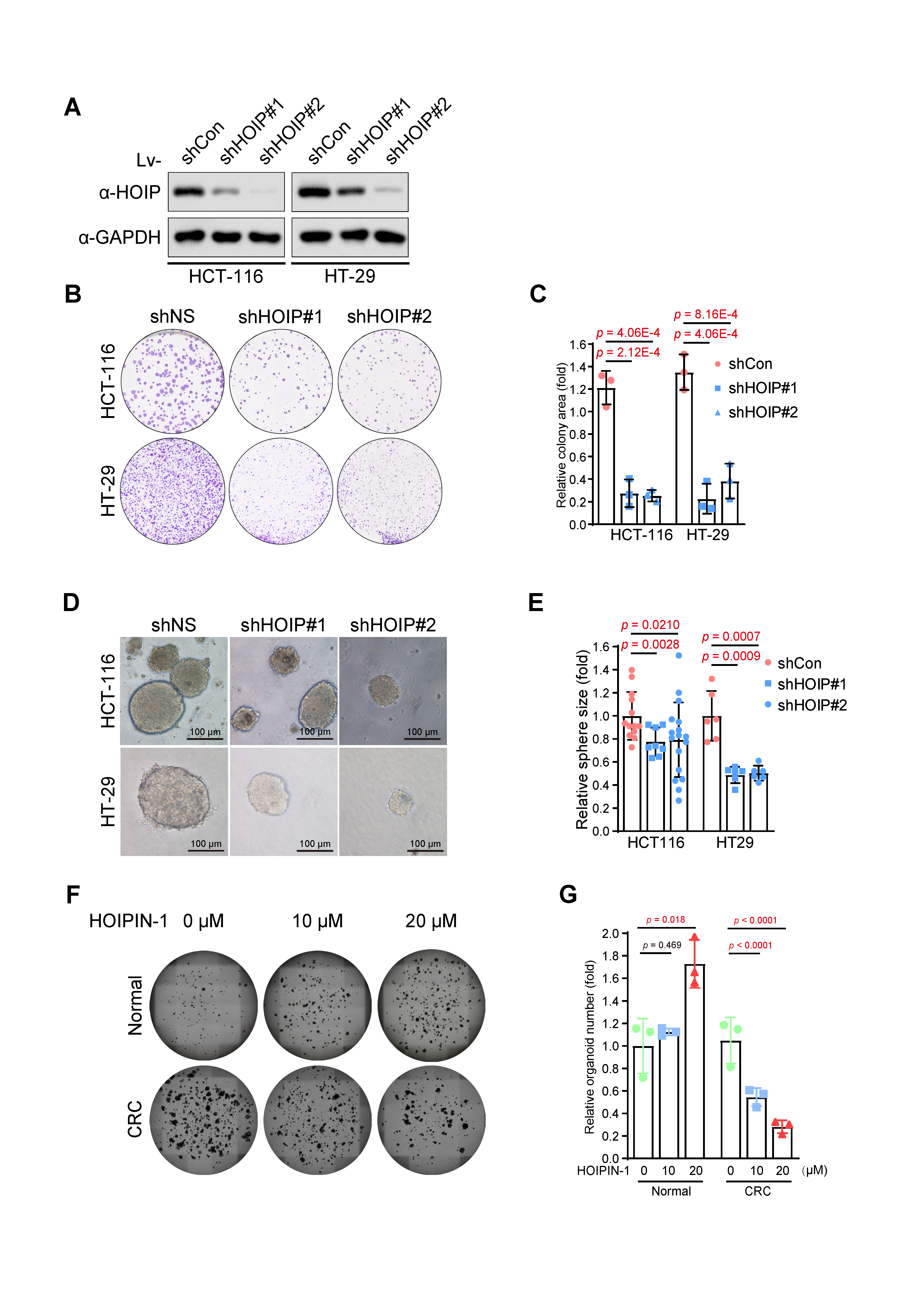

Supplement: Supplementary file 3 — Supplementary Figure 3 [file 41420_2024_2147_MOESM3_ESM.tif]

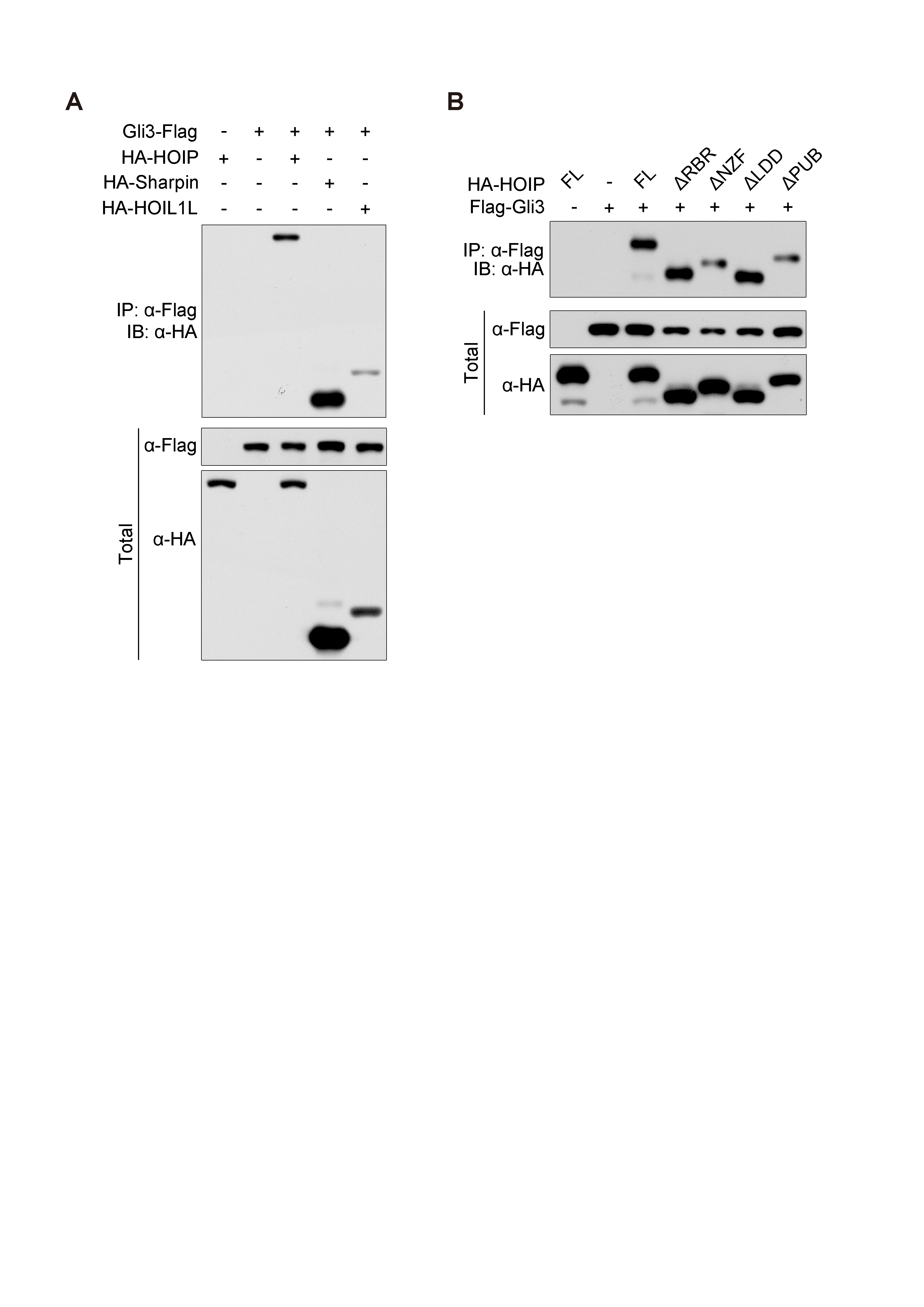

Supplement: Supplementary file 4 — Supplementary Figure 4 [file 41420_2024_2147_MOESM4_ESM.tif]

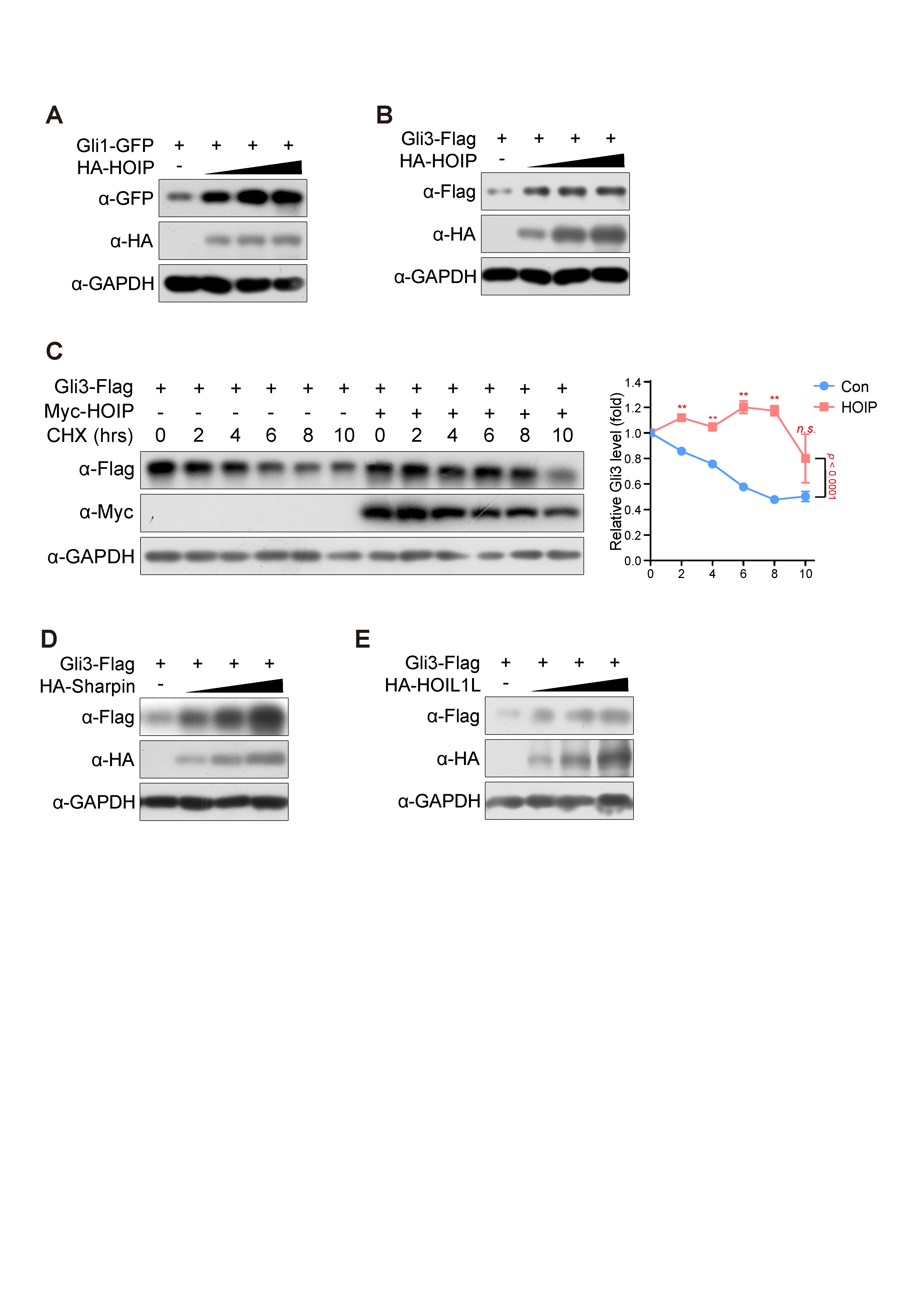

Supplement: Supplementary file 5 — Supplementary Figure 5 [file 41420_2024_2147_MOESM5_ESM.tif]

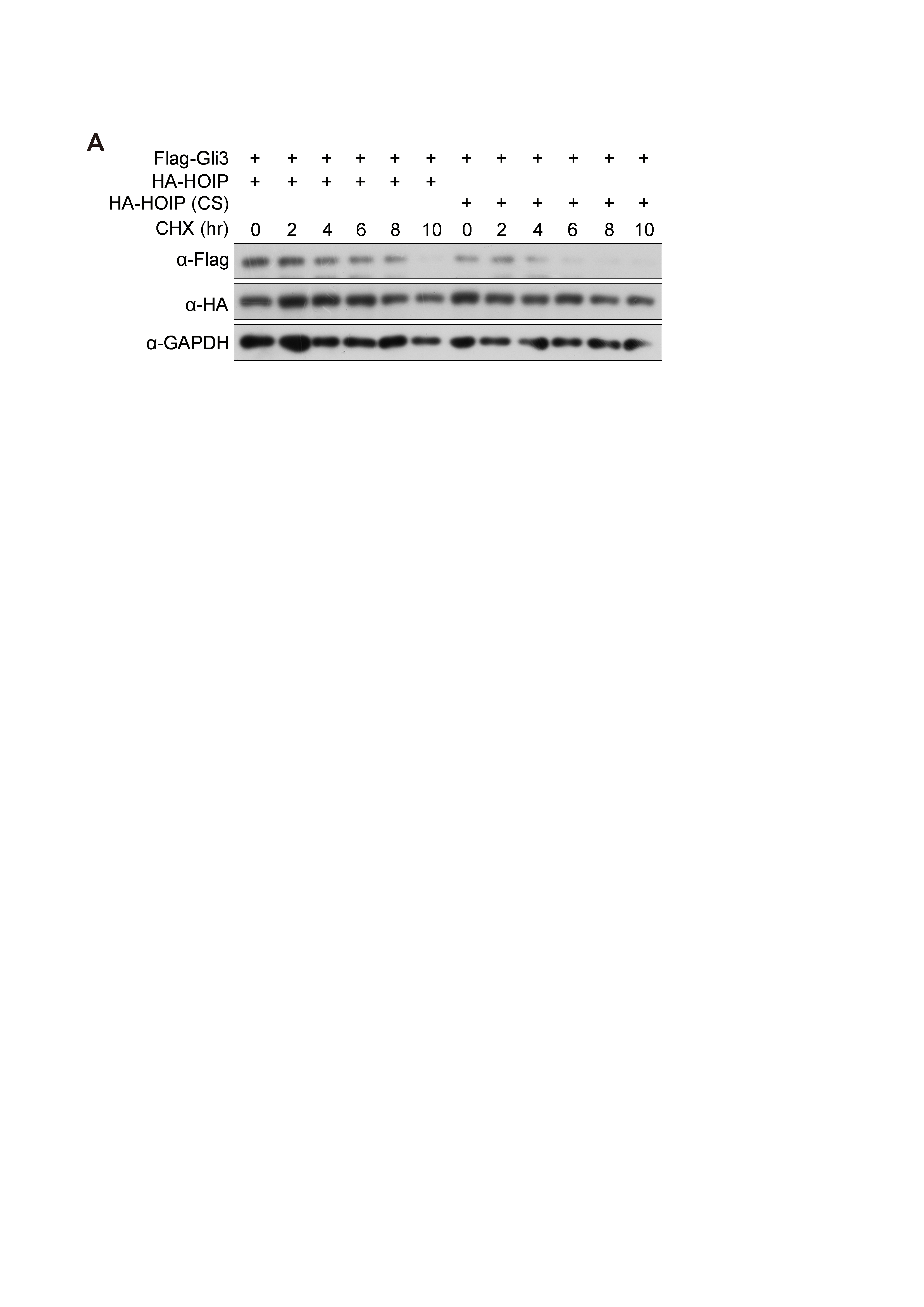

Supplement: Supplementary file 6 — Supplementary Figure 6 [file 41420_2024_2147_MOESM6_ESM.tif]
