# Supplementary material for: Linear polyubiquitylation of Gli protein regulates its protein stability and facilitates tumor growth in colorectal cancer
Source: Cell Death Discov. 2024 Aug 20;10:369. doi: 10.1038/s41420-024-02147-4 (PMC11335874; doi:10.1038/s41420-024-02147-4)
Supplement: Supplementary file 7 — Supplementary Figure Legends [file 41420_2024_2147_MOESM7_ESM.docx]

**Supplementary Figure Legends**

**Supplementary Figure 1. Supplementary to Figure 1.**

(A)-(C) LUBAC is highly expressed in CRC tissues. The expression of each component of LUBAC in the CRC cohort of the TCGA dataset was analysed and visualized via UALCAN (<http://ualcan.path.uab.edu/>).

(D) HOIP correlates with the historical grade of CRC. Representative images of IHC staining of human CRC tissues in different historical grades stained for HOIP.

(E)-(G) Kaplan‒Meier estimates of the overall survival of CRC patients between the low and high expression groups for LUBAC. Expression and survival data of the CRC cohort of the TCGA dataset were downloaded and analysed.

**Supplementary Figure 2. Supplementary to Figure 2.**

(A) Expression validation of the stable cell lines. HCT-116 or SW480 cells stably expressing LV-HOIP were subjected to IB.

(B) Representative images of H&E and IHC staining of xenograft tumours in mice.

**Supplementary Figure 3. Supplementary to Figure 3.**

(A) Expression validation of the stable cell lines. HCT-116 or SW480 cells stably expressing LV-shHOIP were subjected to IB.

(B) HOIP knockdown suppresses the colony formation of CRC cells. HCT-116 or HT-29 cells stably expressing LV-shHOIP were seeded in a 6-well plate for 14 days.

(C) Quantitative analysis of HCT-116 and HT-29 cells stably overexpressing LV-shHOIP. Data are shown as the means ± SDs, n = 3.

(D) HOIP knockdown suppresses the sphere formation of CRC cells. HCT-116 or HT-29 cells stably expressing shHOIP were mixed with soft agar (0.3%) and seeded in a 12-well plate for 14 days.

(E) Quantitative analysis of HCT-116 and HT-29 cells stably overexpressing shHOIP. Data are shown as the means ± SDs.

(F) HOIPIN-1 inhibited the colony formation of PDOs derived from CRC and normal organoids derived from wide-type mouse intestines. Organoids treated with HOIPIN-1 at the indicated concentration were cultured for 11 days or 8 days.

(G) Quantitative analysis of organoids treated with HOIPIN-1. Data are shown as the means ± SDs, n = 3.

**Supplementary Figure 4. Supplementary to Figure 4.**

(A) Gli3 interacts with LUBAC components. HEK-293T cells transfected with HA-HOIP, Sharping or HOIL and Flag-Gli3 plasmids were subjected to a co-IP assay.

(B) HOIP binds to Gli3 through its PUB domain. HEK-293T cells transfected with Flag-Gli3 and HA-HOIP or its truncated mutation plasmids were subjected to a co-IP assay.

**Supplementary Figure 5. Supplementary to Figure 5.**

(A)-(B) HOIP elevates the protein levels of exogenous Gli1 and Gli3. HEK-293T cells transfected with Flag-Gli1 **(A)** or Gli3 **(B)** and HA-HOIP plasmids were subjected to IB with the indicated antibodies.

(C) HOIP impairs the degradation of Gli3. Cycloheximide (CHX) (100 μg/ml) was incubated for the indicated period with HEK-293T cells transfected with or without Flag-Gli3 and HA-HOIP. Cell lysates were harvested for IB with the indicated antibody.

(D)-(E) LUBAC stabilizes Gli3. HEK-293T cells transfected with Flag-Gli3 and HA-Sharpin **(D)** or HOIL **(E)** plasmids were subjected to IB with the indicated antibodies.

**Supplementary Figure 6. Supplementary to Figure 6.**

(A) A HOIP mutant with loss of its E3 ligase activity cannot stabilize Gli3. Cycloheximide (CHX) (100 μg/ml) was incubated for the indicated period with HEK-293T cells transfected with Flag-Gli3 and HA-HOIP or its point mutation. Cell lysates were harvested for IB with the indicated antibody.
