# Supplementary material for: Linear polyubiquitylation of Gli protein regulates its protein stability and facilitates tumor growth in colorectal cancer
Source: Cell Death Discov. 2024 Aug 20;10:369. doi: 10.1038/s41420-024-02147-4 (PMC11335874; doi:10.1038/s41420-024-02147-4)
Supplement: Supplementary file 8 — Supplementary Table 1 [file 41420_2024_2147_MOESM8_ESM.docx]

**Supplementary Table 1.** Antibodies.

| Target protein | Supplier | Cat. No. | Usage |
| --- | --- | --- | --- |
| Gli1 | Cell Signaling Technology | 2643s | IB |
| Gli2 | Santa Cruz Biotechnology | sc-271786 | IB and co-IP |
| Gli3 | Novus Biologicals | NBP2-29627 | IB and co-IP |
| HOIP | Abcam | ab46322 | IB and IHC |
| GAPDH | Proteintech | 60004-1-Ig | IB |
| Flag (tag) | Sigma-Aldrich | F3165-1MG | IB and co-IP |
| HA (tag) | Proteintech | 66006-2-Ig | IB |
| MYC (tag) | Proteintech | 60003-2-Ig | IB |
| Ubiquitin | Santa Cruz Biotechnology | sc-8017 | IB |
| M1-ubiquitin chain | Merck | MABS451 | IB |
| Ki67 | Proteintech | 28074-1-AP | IHC |
