# Supplementary material for: Linear polyubiquitylation of Gli protein regulates its protein stability and facilitates tumor growth in colorectal cancer
Source: Cell Death Discov. 2024 Aug 20;10:369. doi: 10.1038/s41420-024-02147-4 (PMC11335874; doi:10.1038/s41420-024-02147-4)
Supplement: Supplementary file 9 — Supplementary Table 2 [file 41420_2024_2147_MOESM9_ESM.docx]

**Supplementary Table 2.** Primers.

| Primer | Sequence | Target | Usage |
| --- | --- | --- | --- |
| shHOIP#1-f | gatccccGCAGAAGGAGGTTCCCAATttcaagagaATTGGGAACCTCCTTCTGCttttta | HOIP | Generating shRNA |
| shHOIP#1-r | agcttaaaaaGCAGAAGGAGGTTCCCAATtctcttgaaATTGGGAACCTCCTTCTGCggg |  |  |
| shHOIP#2-f | gatccccGCATGAACGACCCAGAATAttcaagagaTATTCTGGGTCGTTCATGCttttta |  |  |
| shHOIP#2-r | agcttaaaaaGCATGAACGACCCAGAATAtctcttgaaTATTCTGGGTCGTTCATGCggg |  |  |
| Gapdh-f | AAGAAGGTGGTGAAGCAG | *Gapdh* | qPCR |
| Gapdh-r | TCATACCAGGAAATGAGC |  |  |
| Ascl2-f | GCCTGACCAAATGCCAAGTG | *Ascl2* |  |
| Ascl2-r | ATTTCCAAGTCCTGATGCTGC |  |  |
| Axin2-f | GCTCCAGAAGATCACAAAGAGC | *Axin2* |  |
| Axin2-r | AGCTTTGAGCCTTCAGCATC |  |  |
| cmyc-f | GCTGTTTGAAGGCTGGATTTC | *c-myc* |  |
| cmyc-r | GATGAAATAGGGCTGTACGGAG |  |  |
