# Supplementary material for: Linear polyubiquitylation of Gli protein regulates its protein stability and facilitates tumor growth in colorectal cancer
Source: Cell Death Discov. 2024 Aug 20;10:369. doi: 10.1038/s41420-024-02147-4 (PMC11335874; doi:10.1038/s41420-024-02147-4)

Figure 1 C

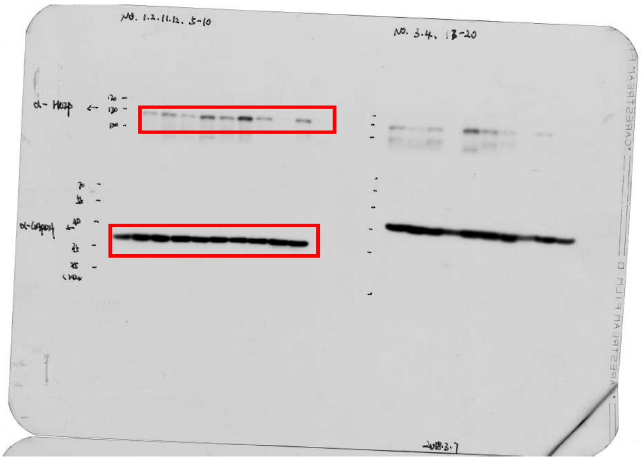

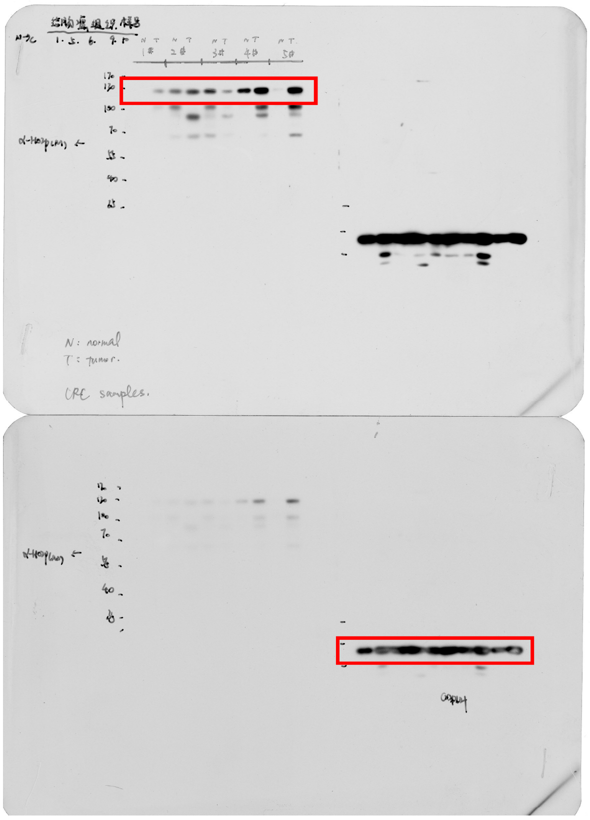

Supplementary Figure 2A

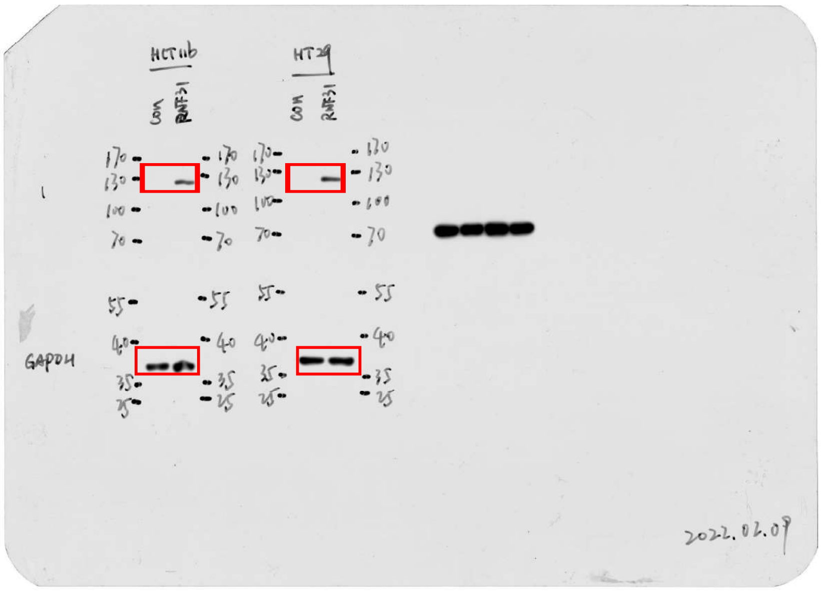

Supplementary Figure 3A

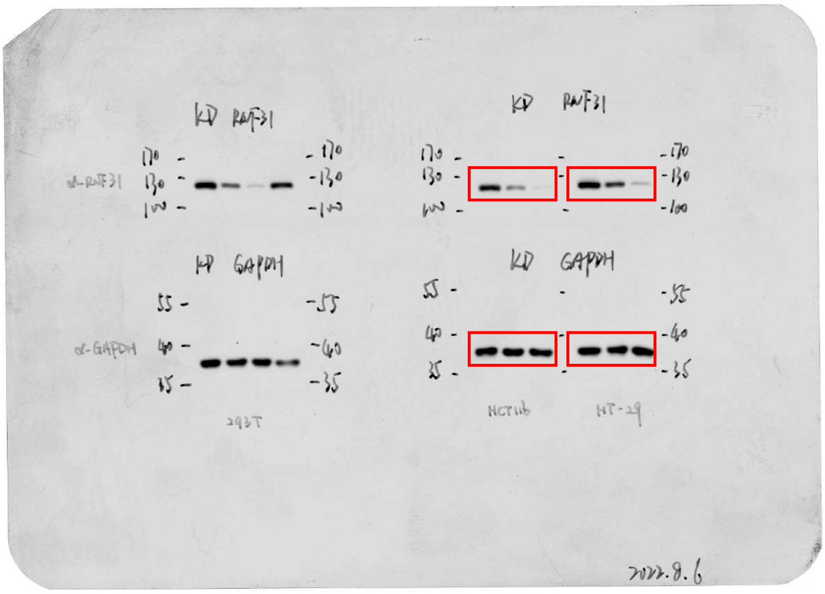

Figure 4 A

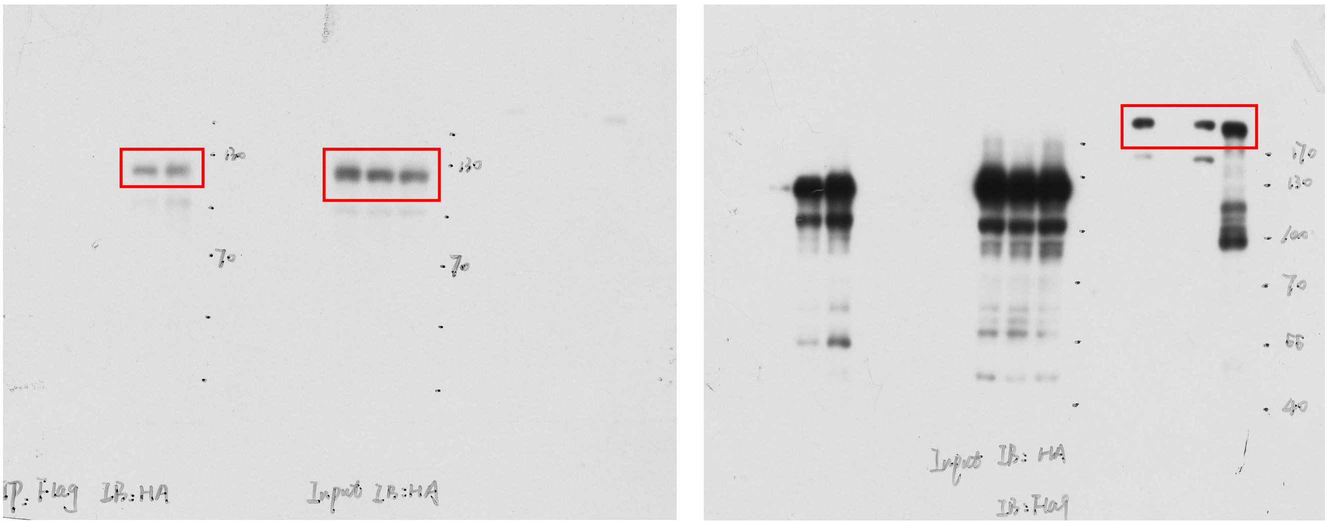

Figure 4 B

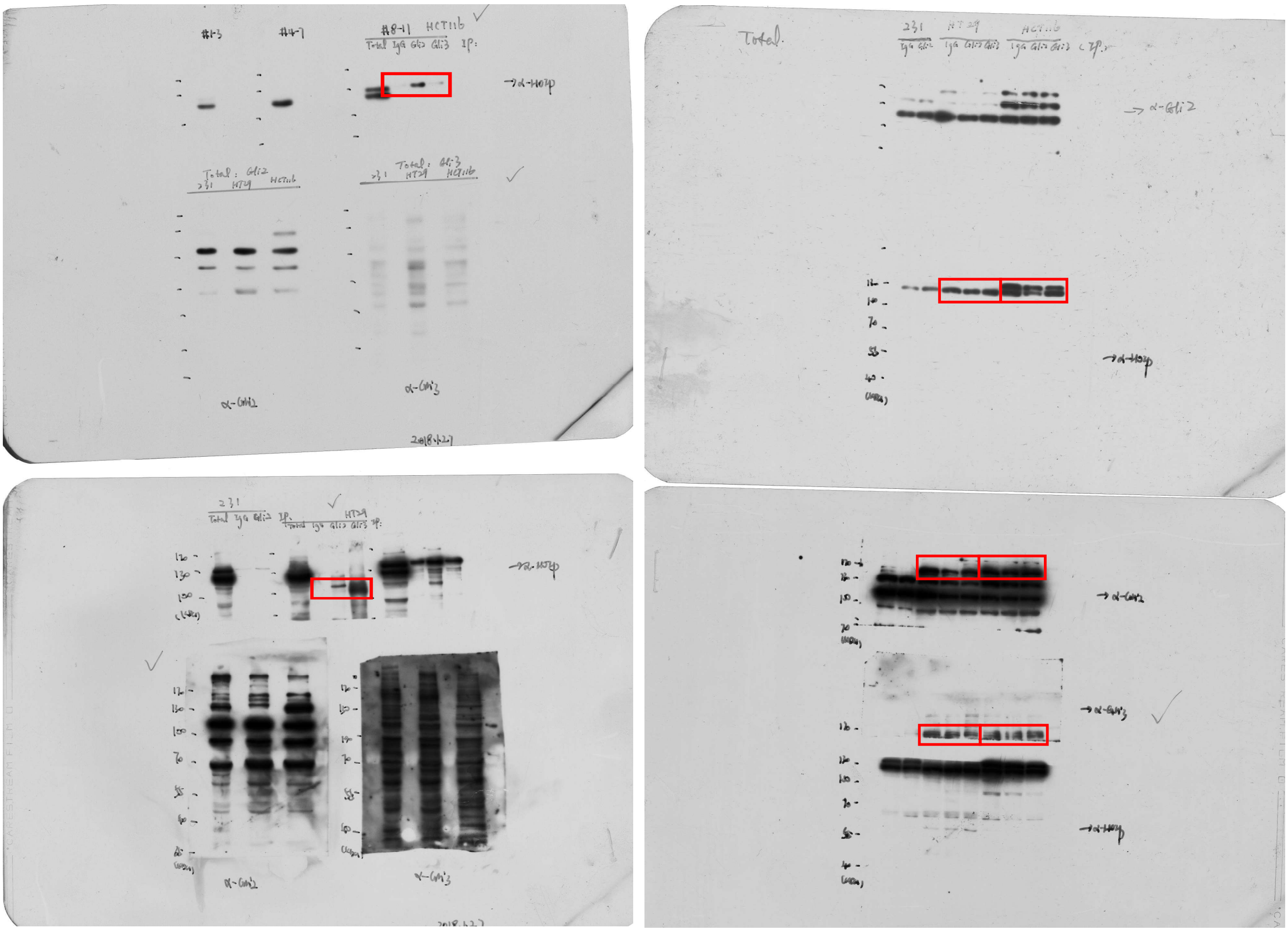

Figure 4 C

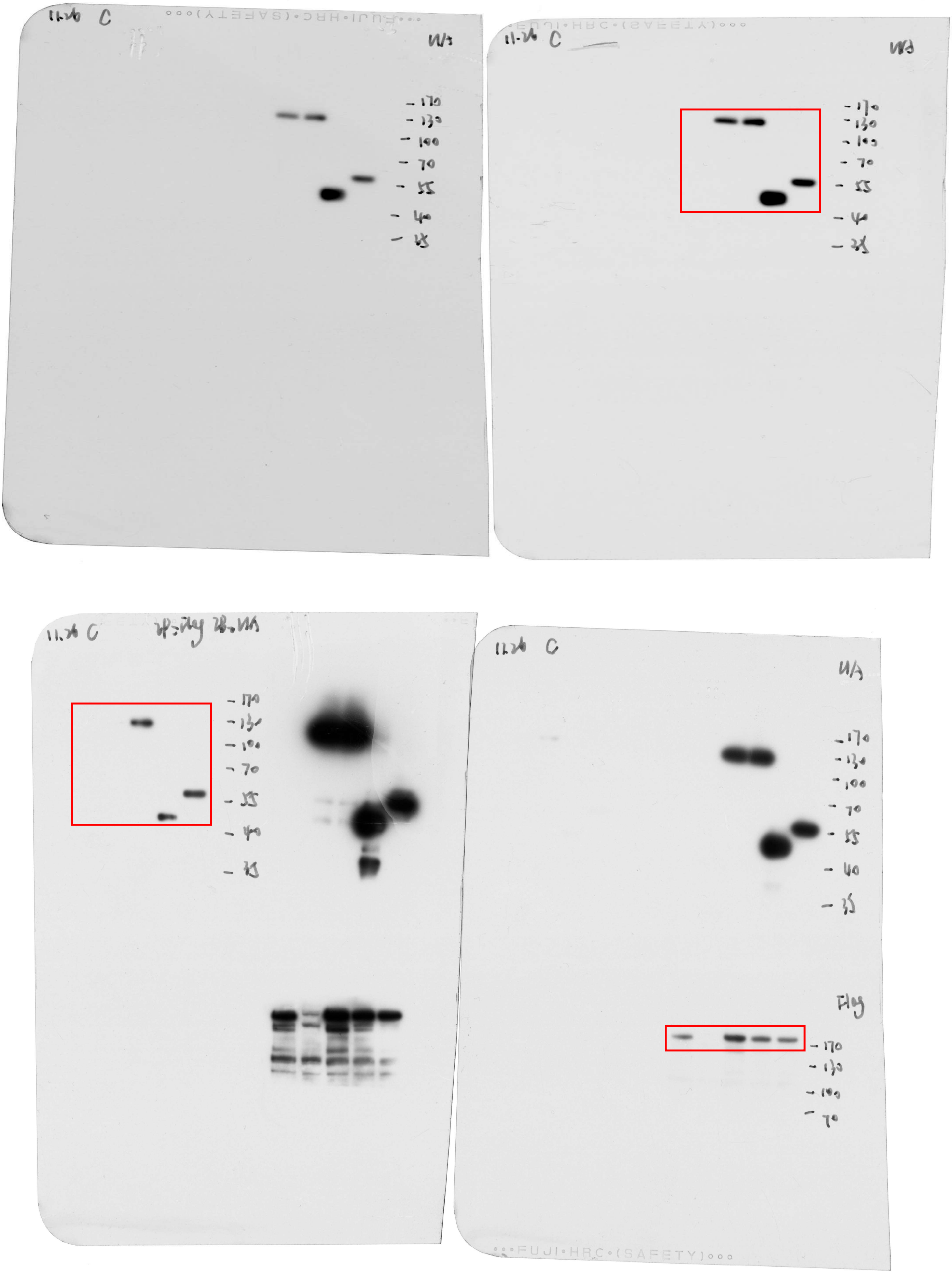

Figure 4E

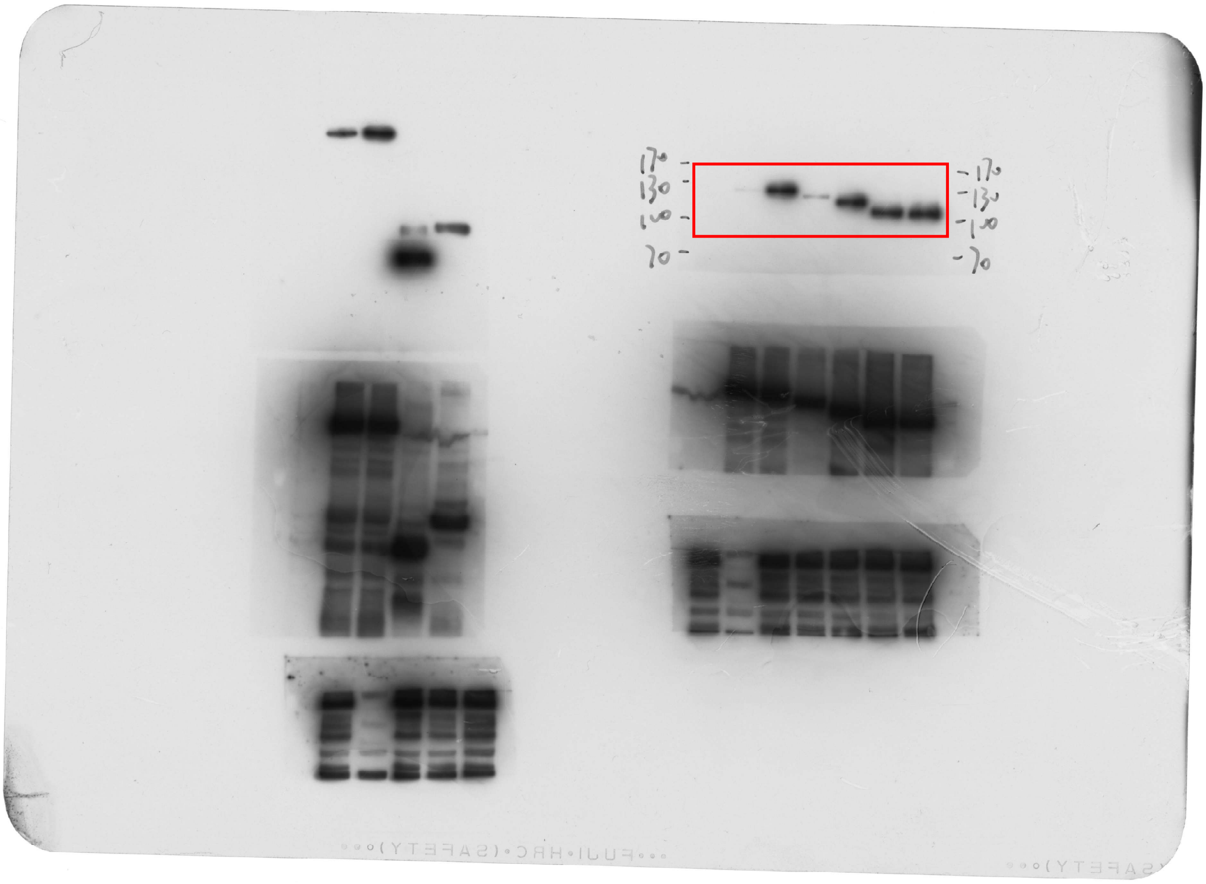
170
130
100
70
170
130
100
70

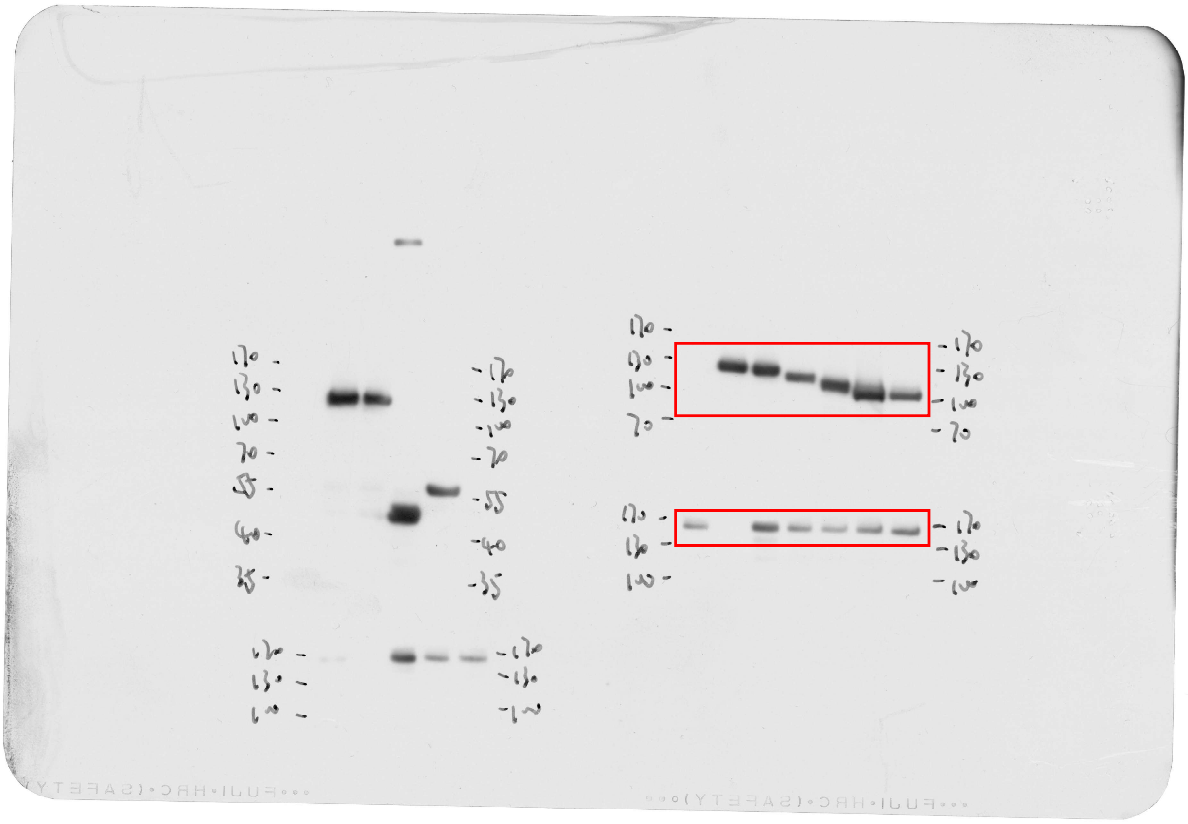
170
130
100
70
55
40
35
170
130
100
70
55
40
35
170
130
100
170
130
100
170
130
100
70
170
130
100
70
170
130
100
170
130
100

Figure 4F

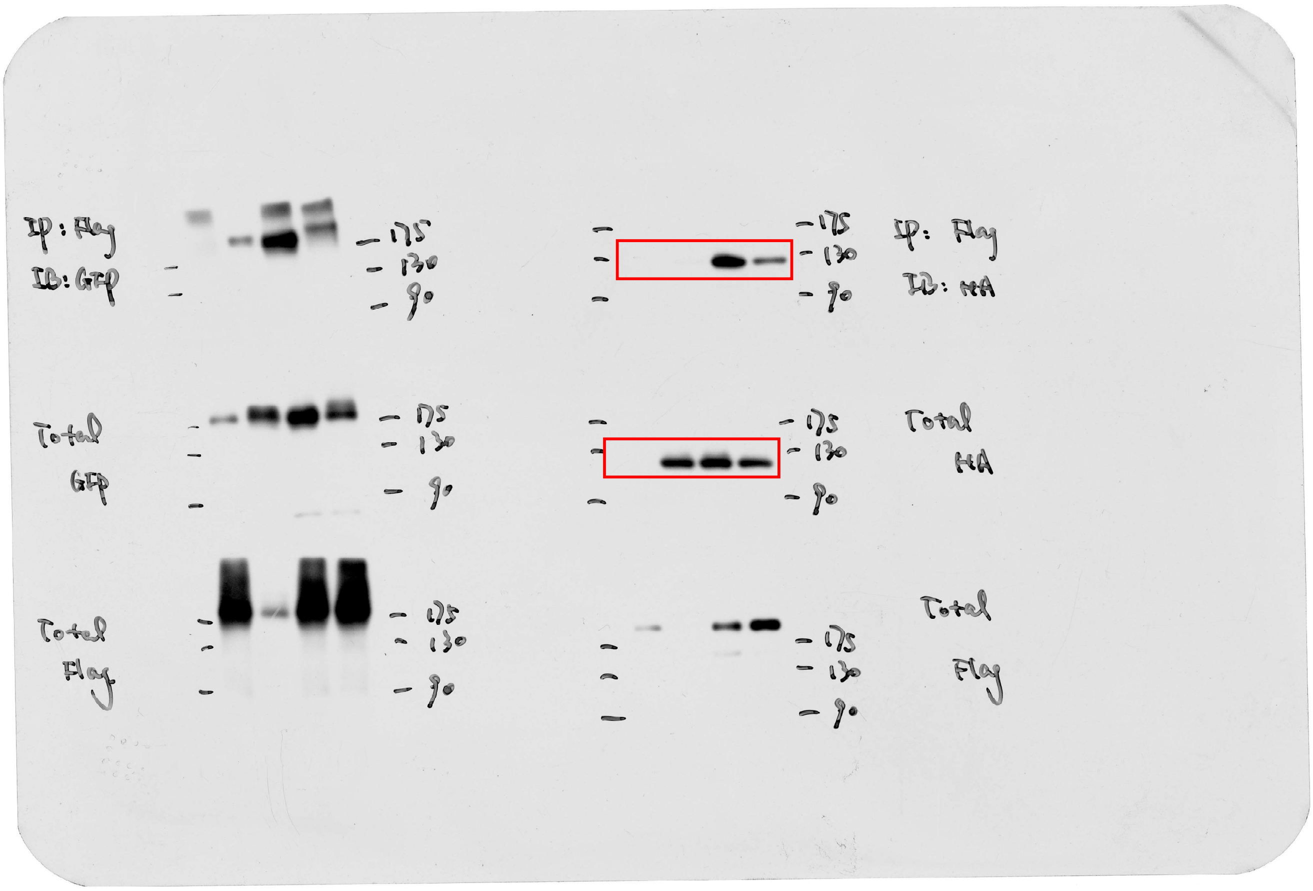
IP: Flag
IB: GFP
175
130
90
Total
GFP
Total
Flag
IP: Flag
IB: HA
Total
HA
Total
Flag

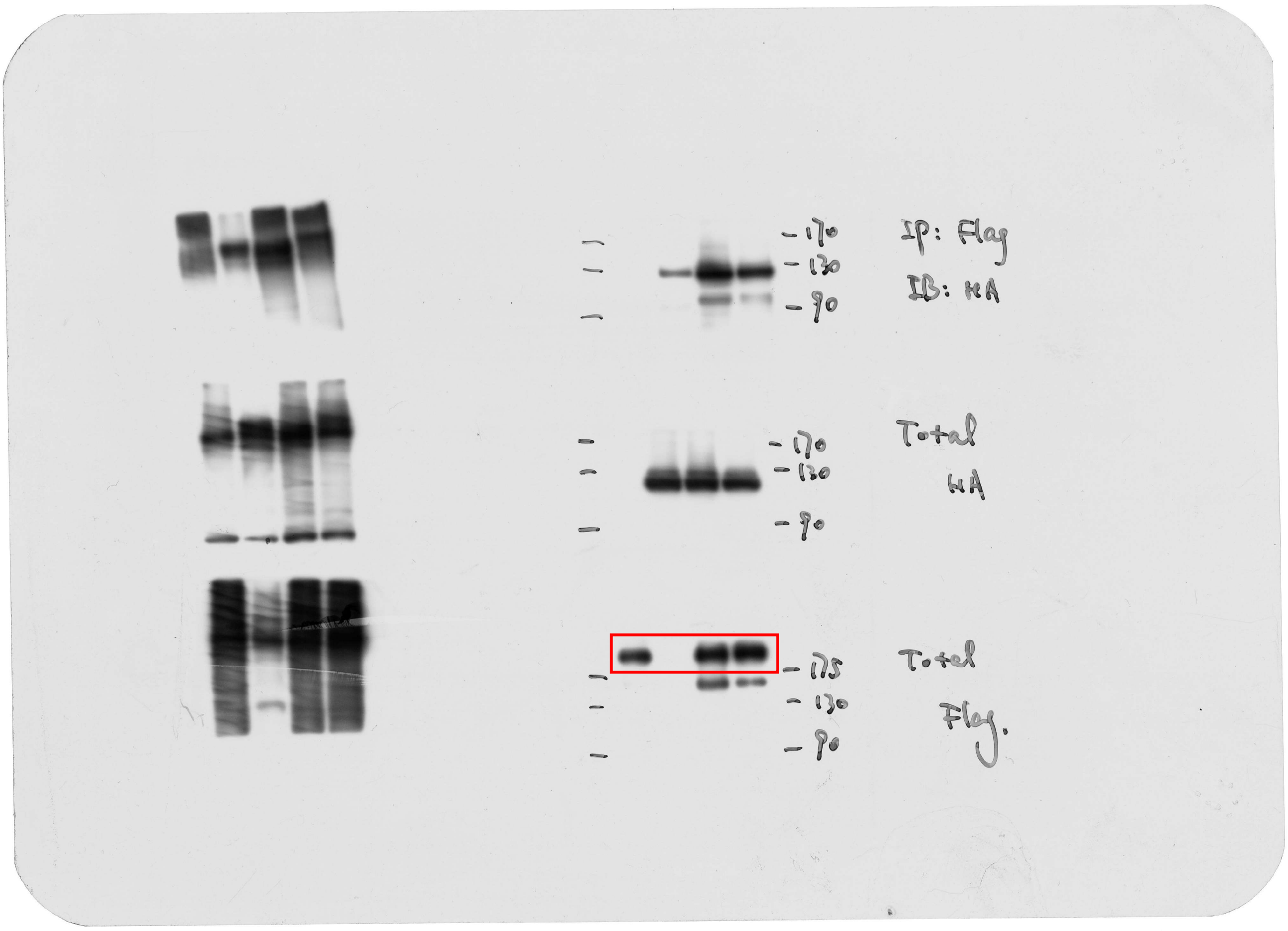
IP: Flag
IB: HA
170
130
90
Total
HA
175
Total
Flag.

Supplementary Figure 4A

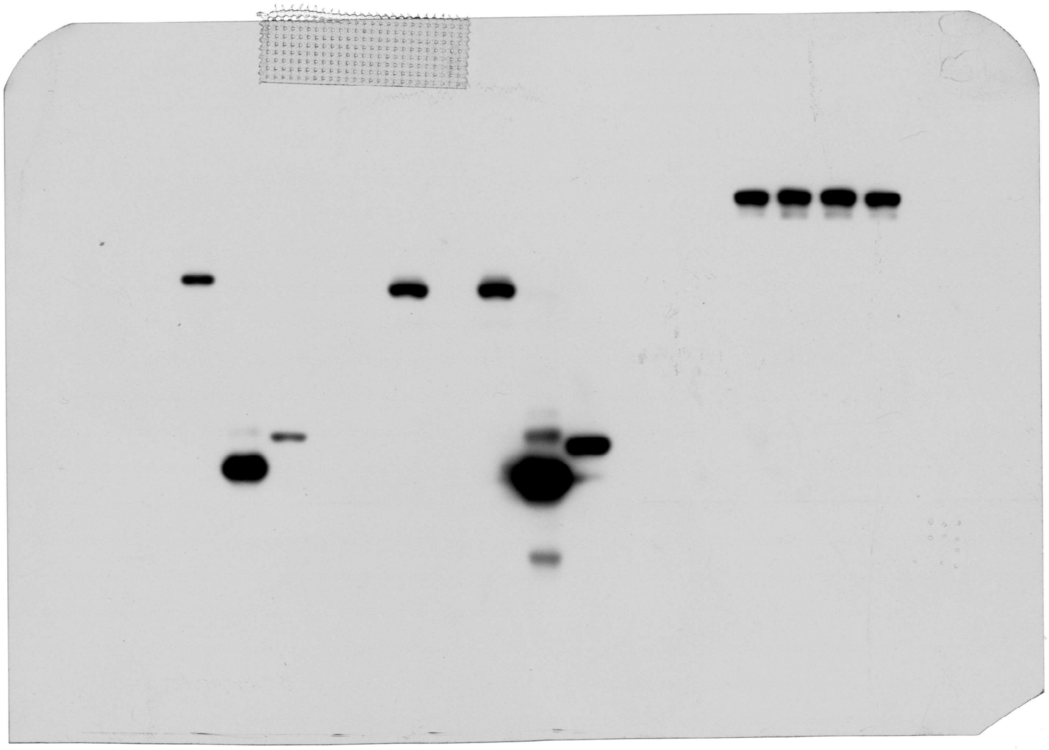

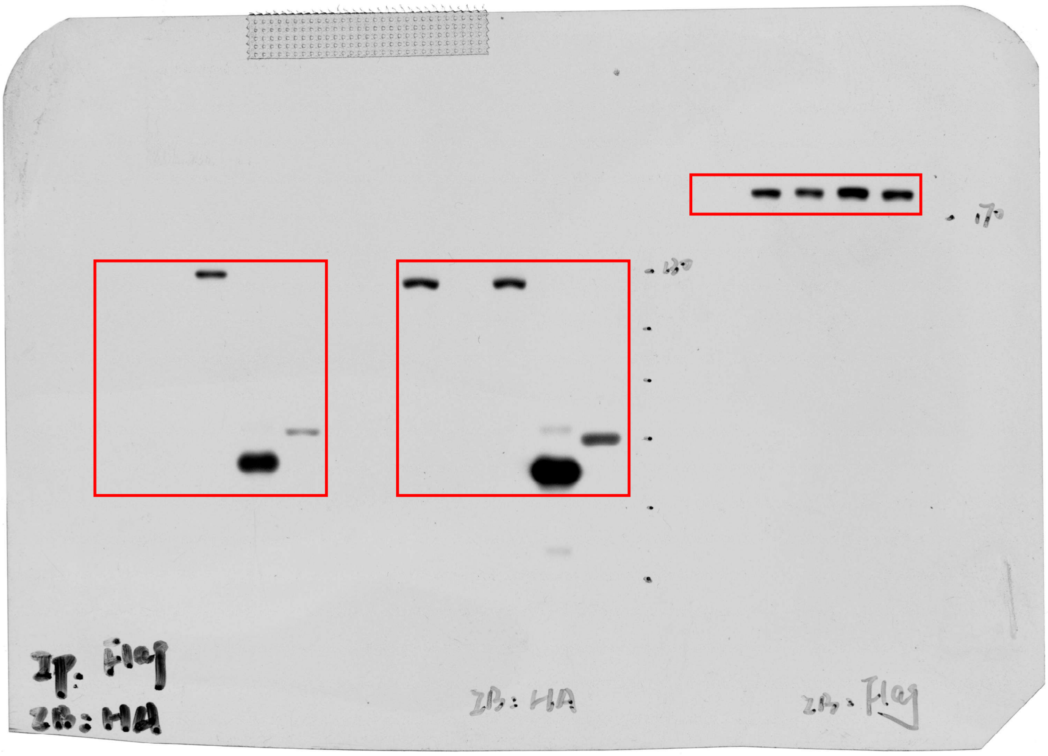

Supplementary Figure 4B

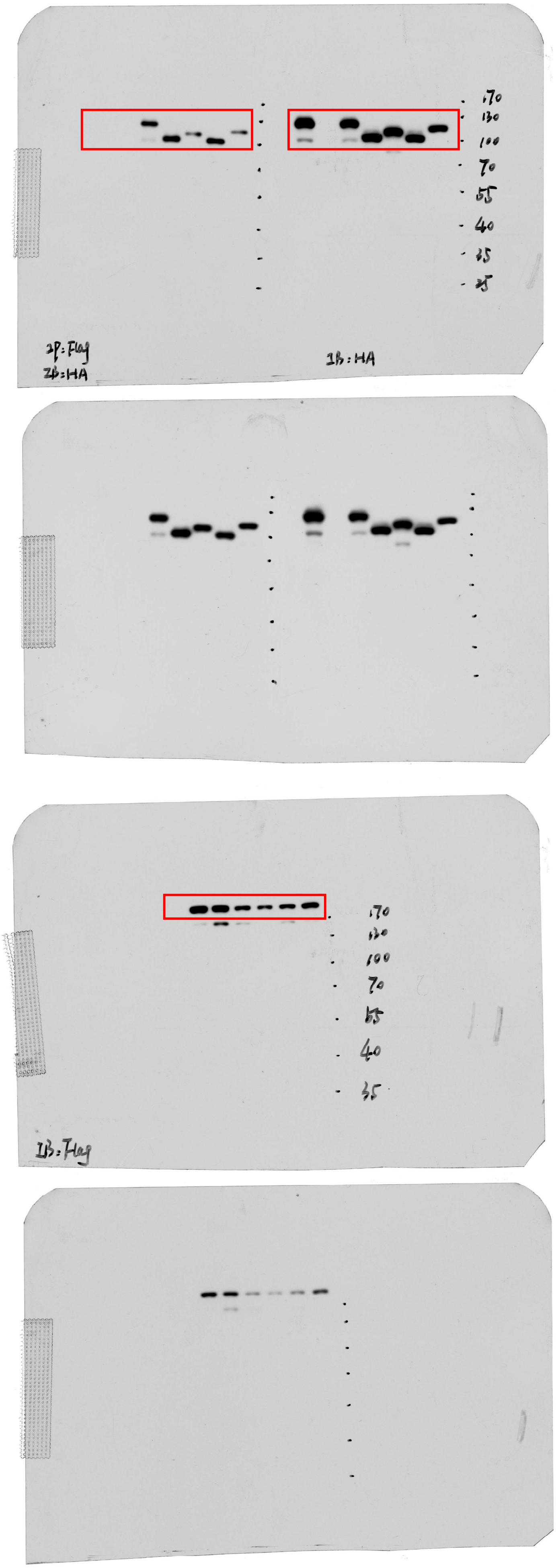

Figure 5 B

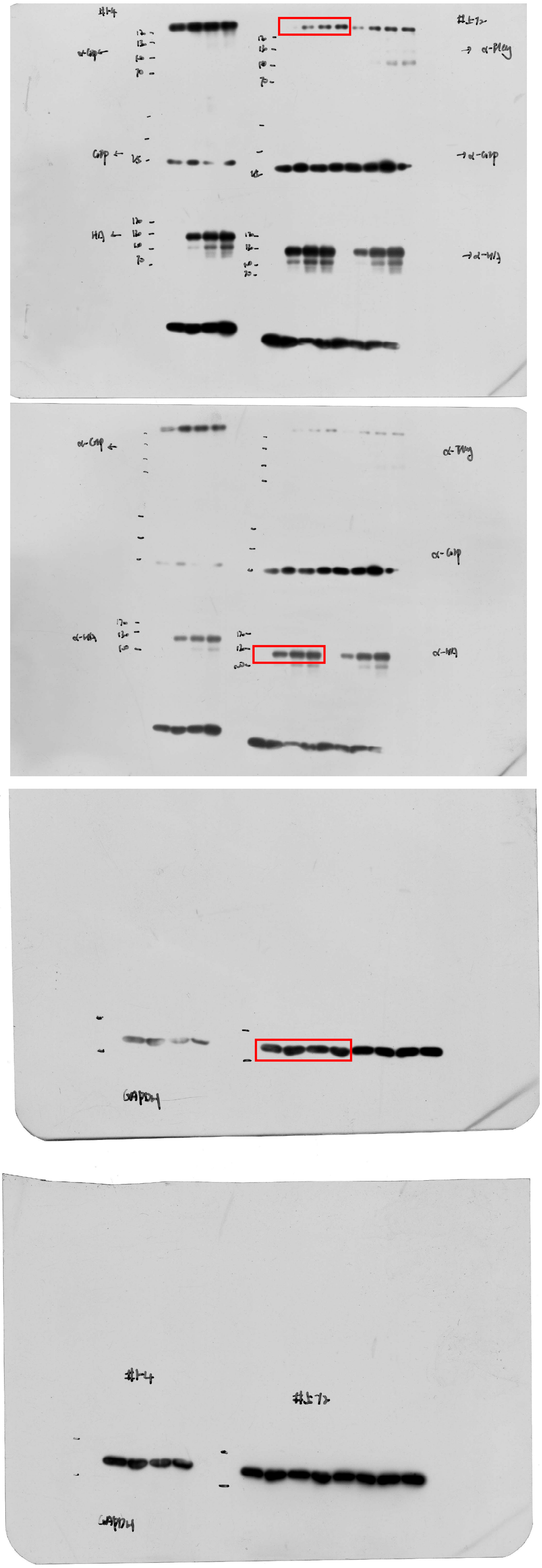

Figure 5 C

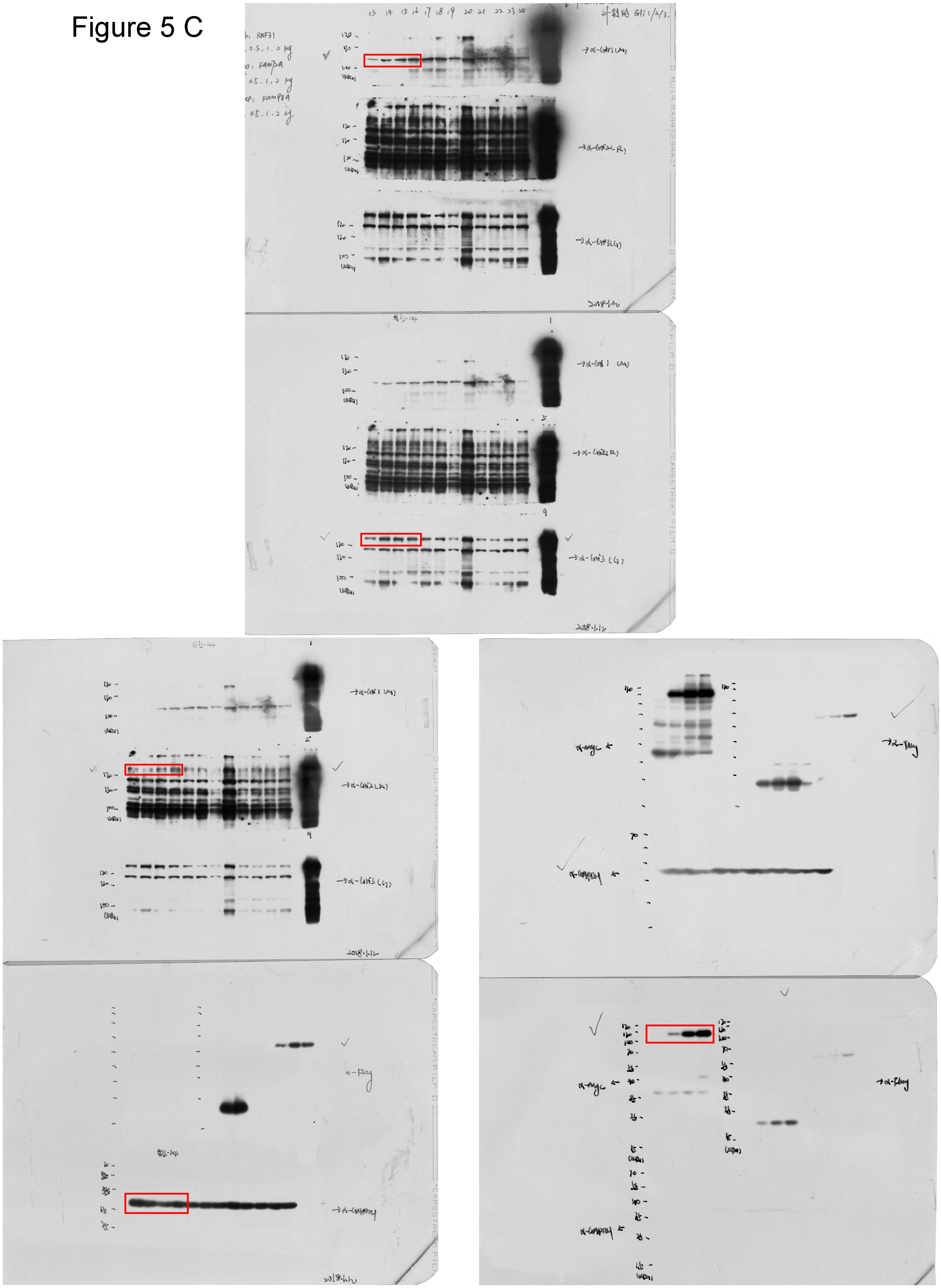

Figure 5 F 5G

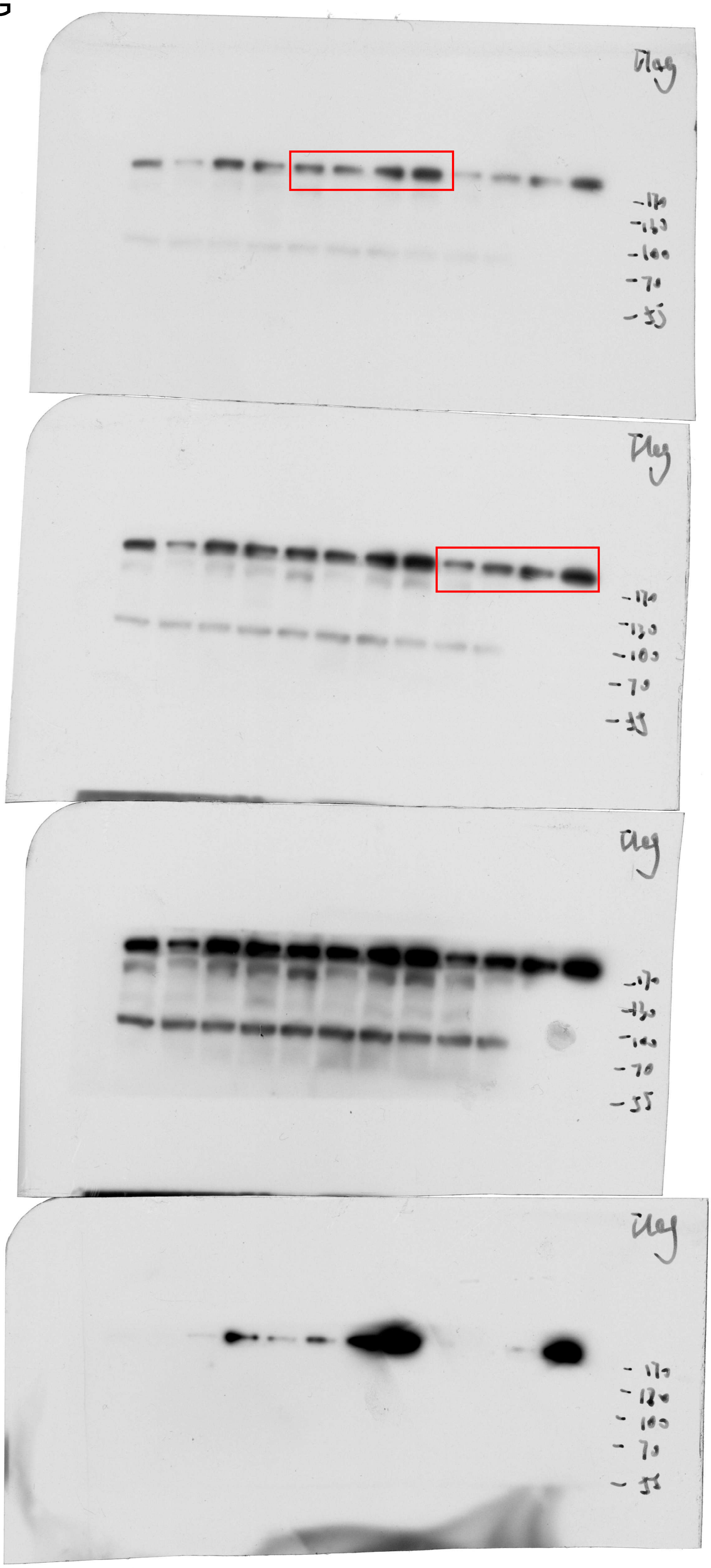

Figure 5 F G

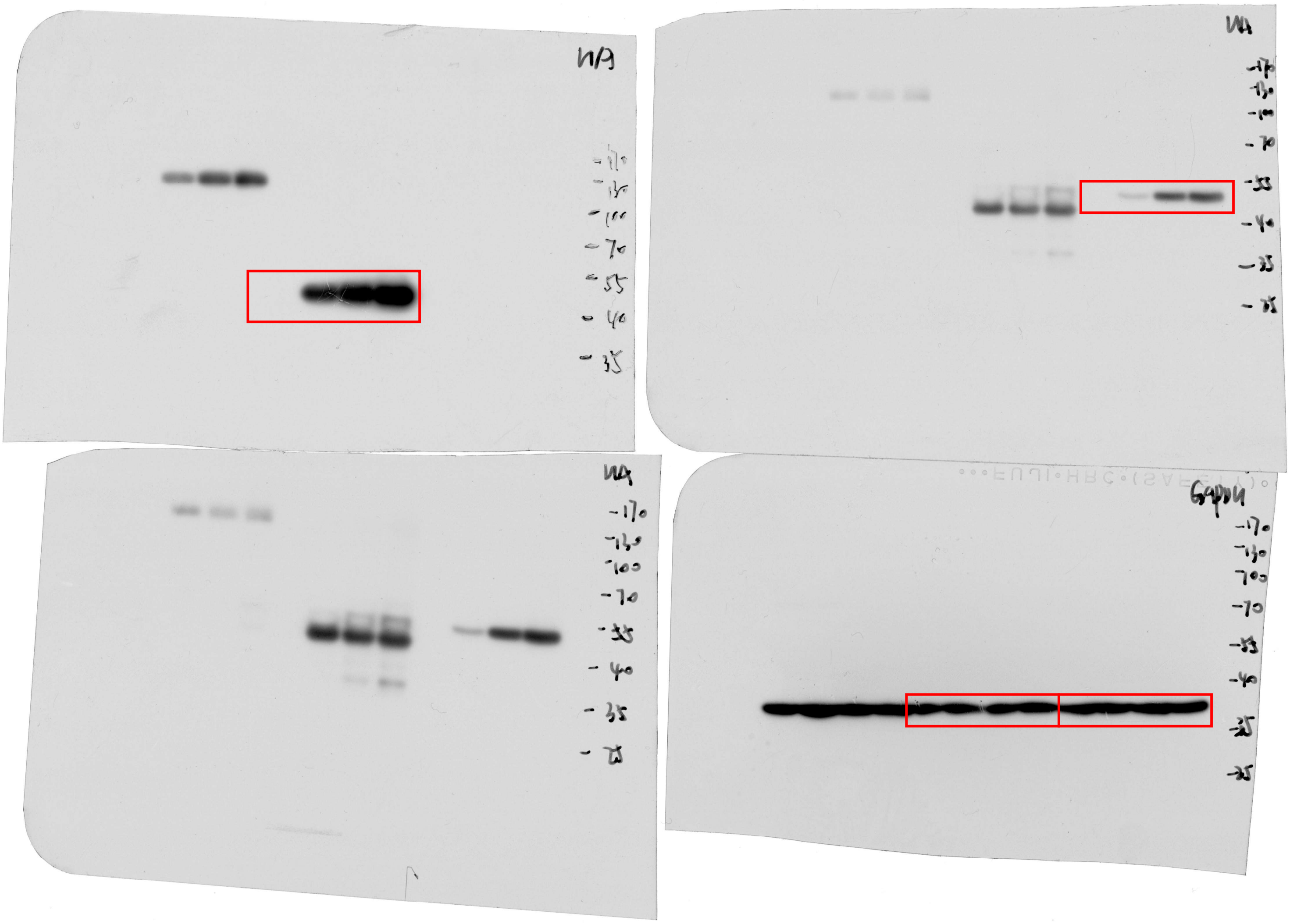

Supplementary Figure 5A

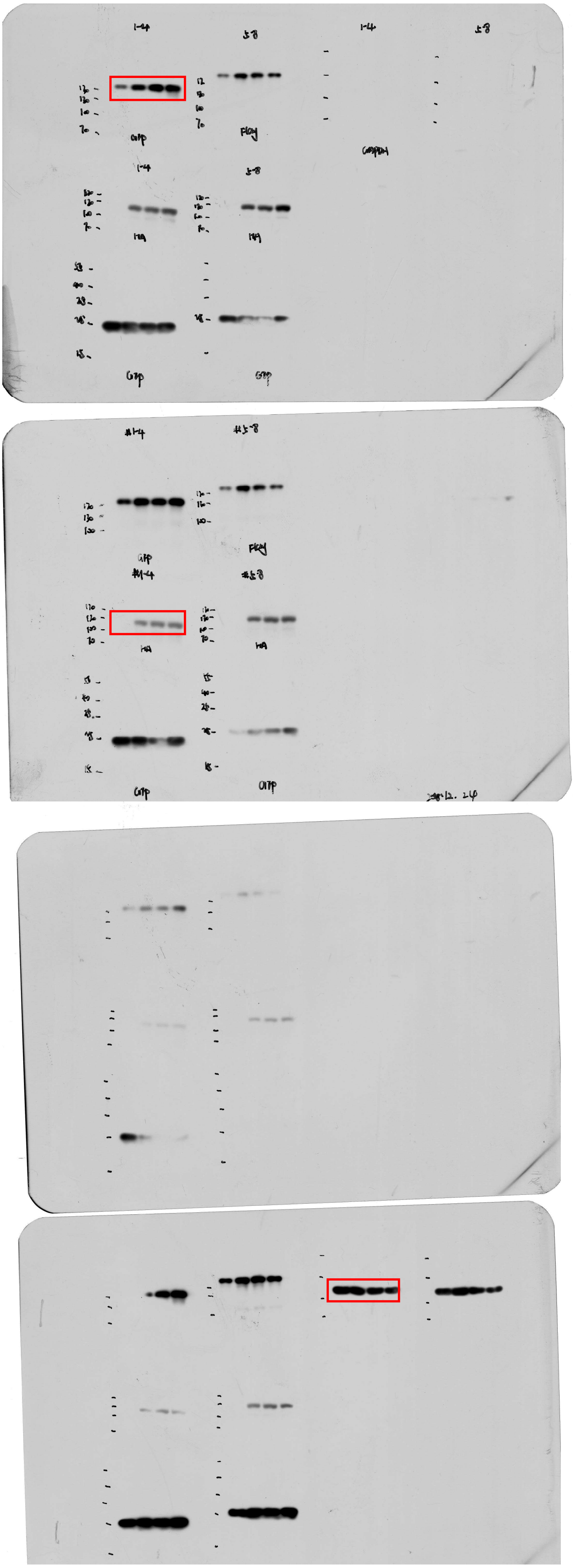

Supplementary Figure 5B

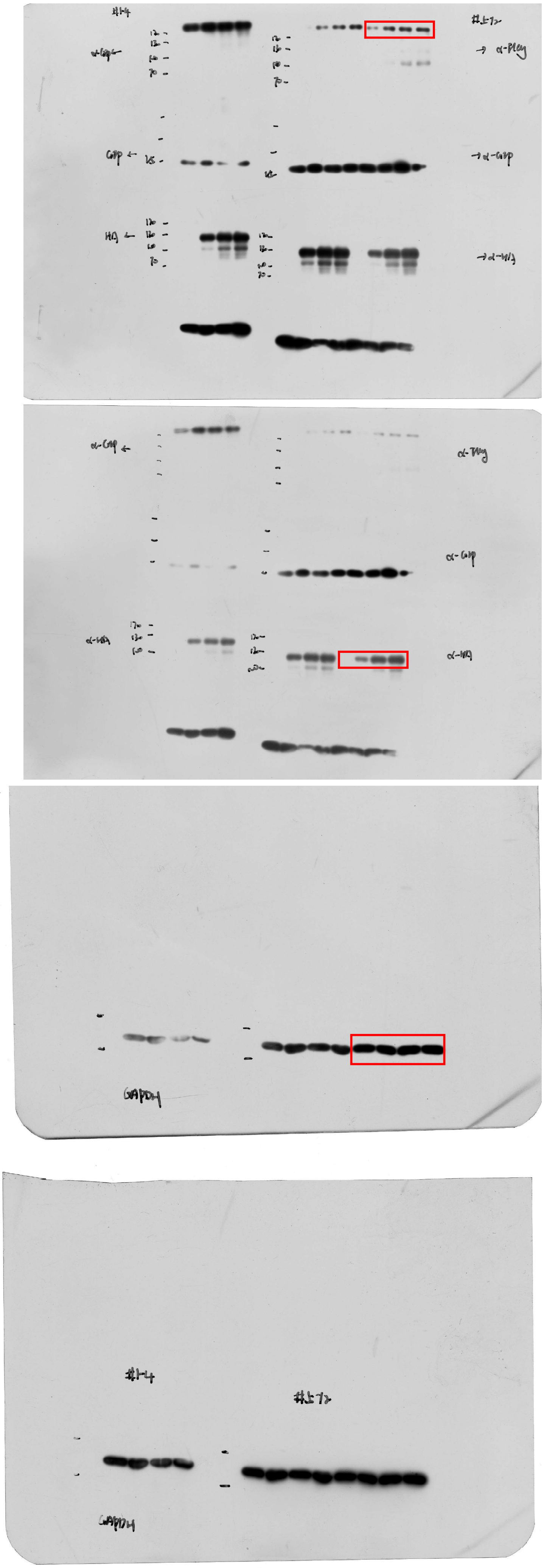

Supplementary Figure 5C

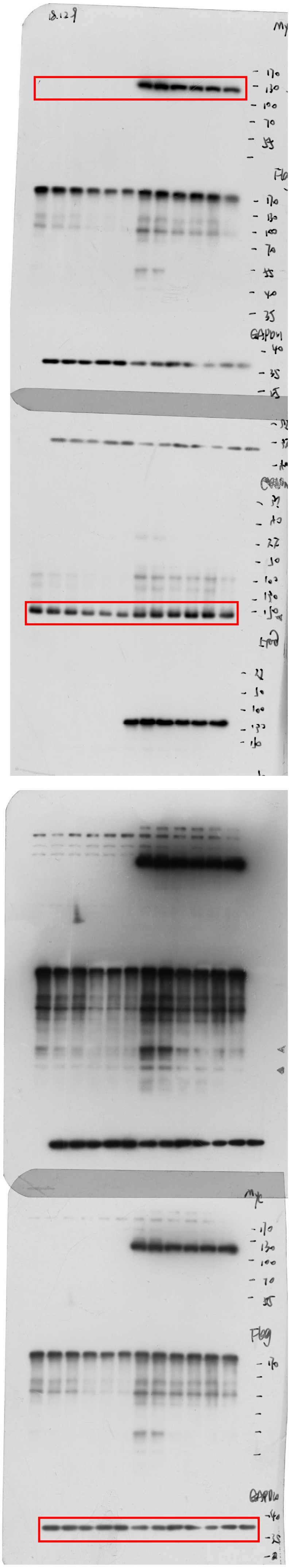

Supplementary Figure 5D

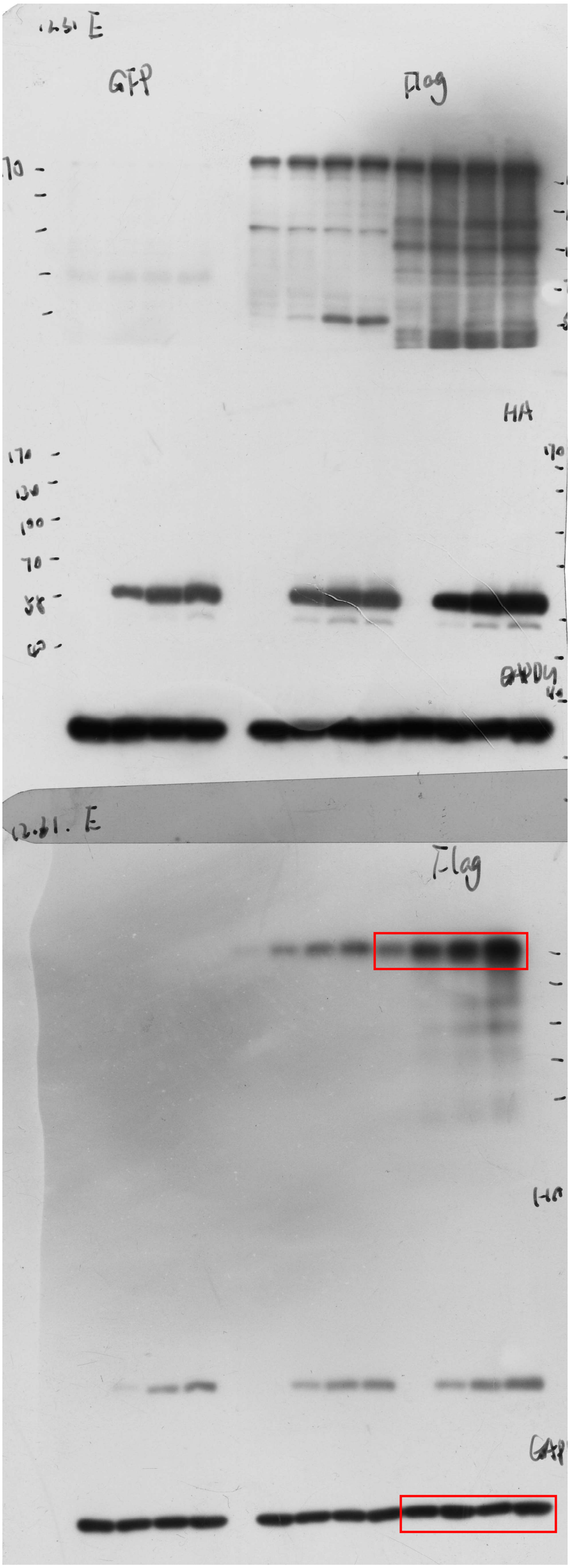

Supplementary Figure 5E

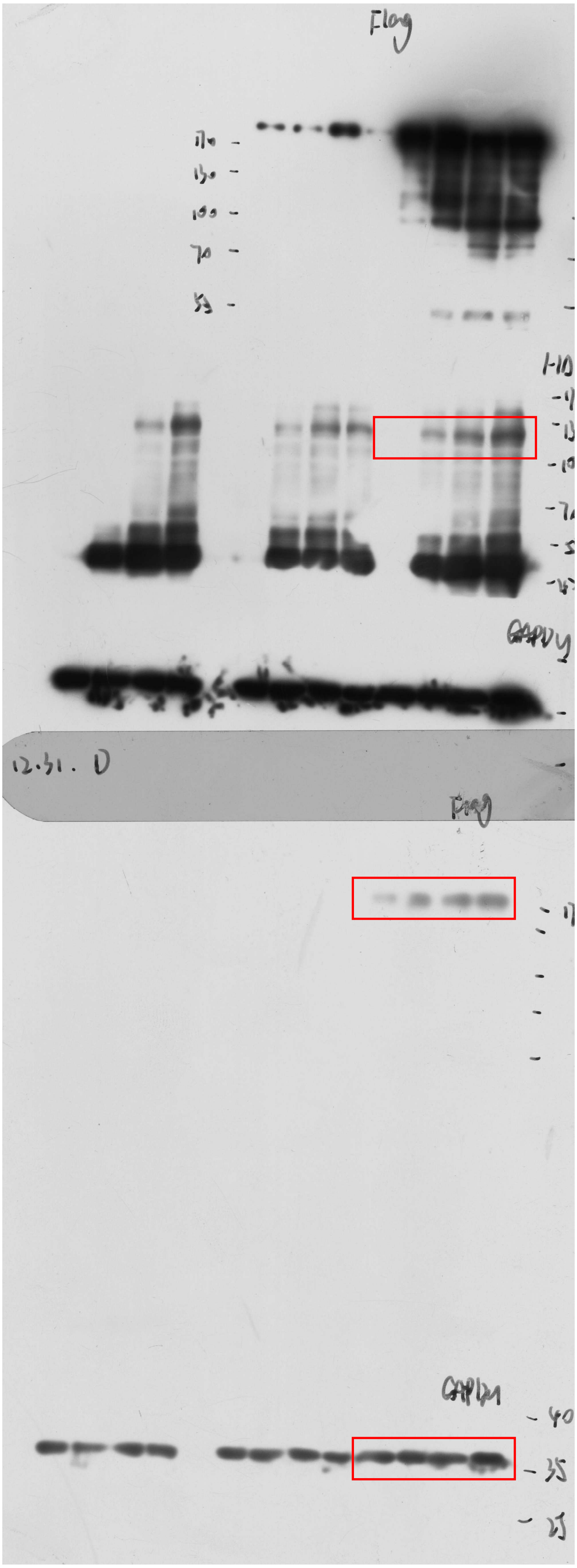

Figure 6 A (Left)

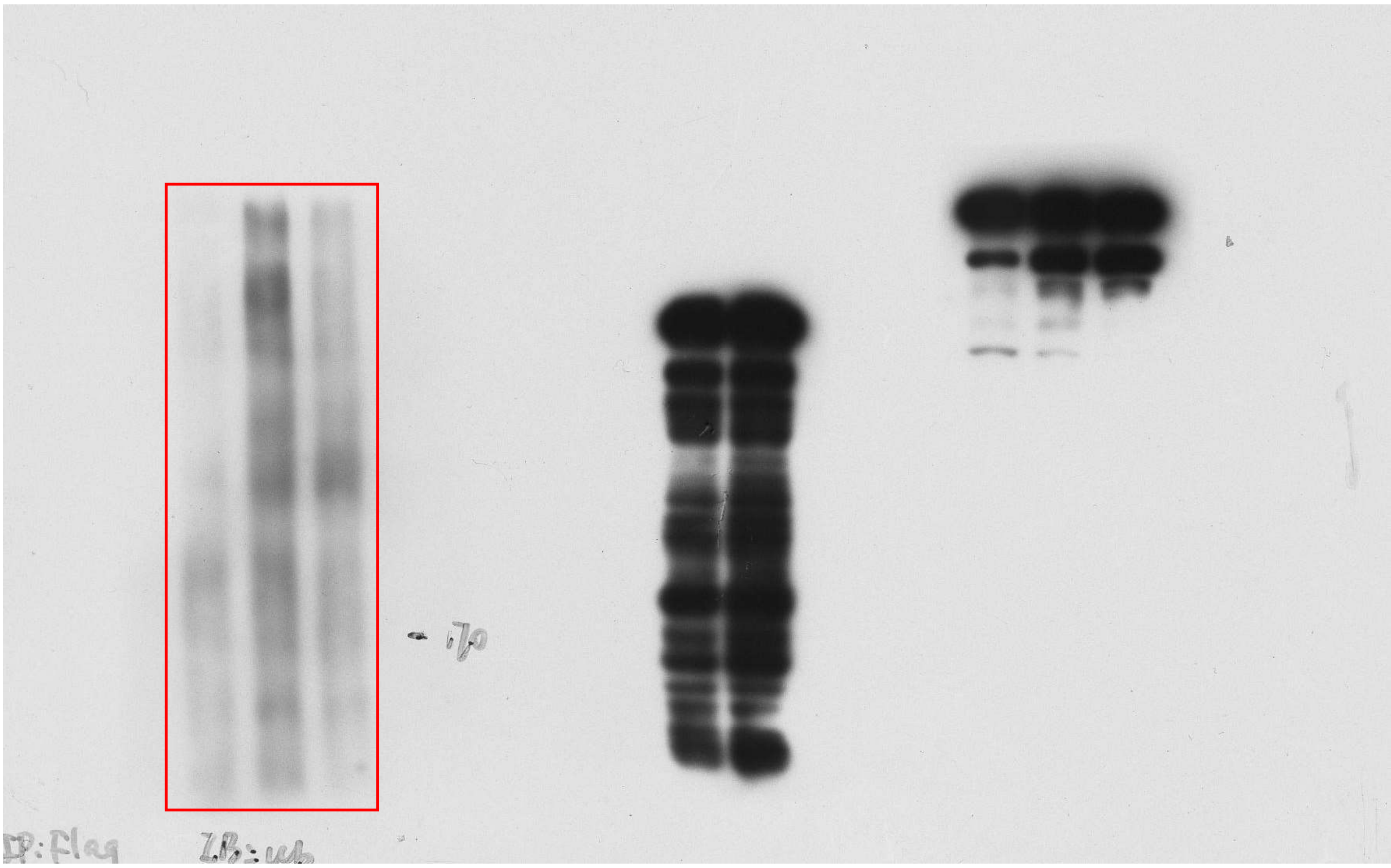

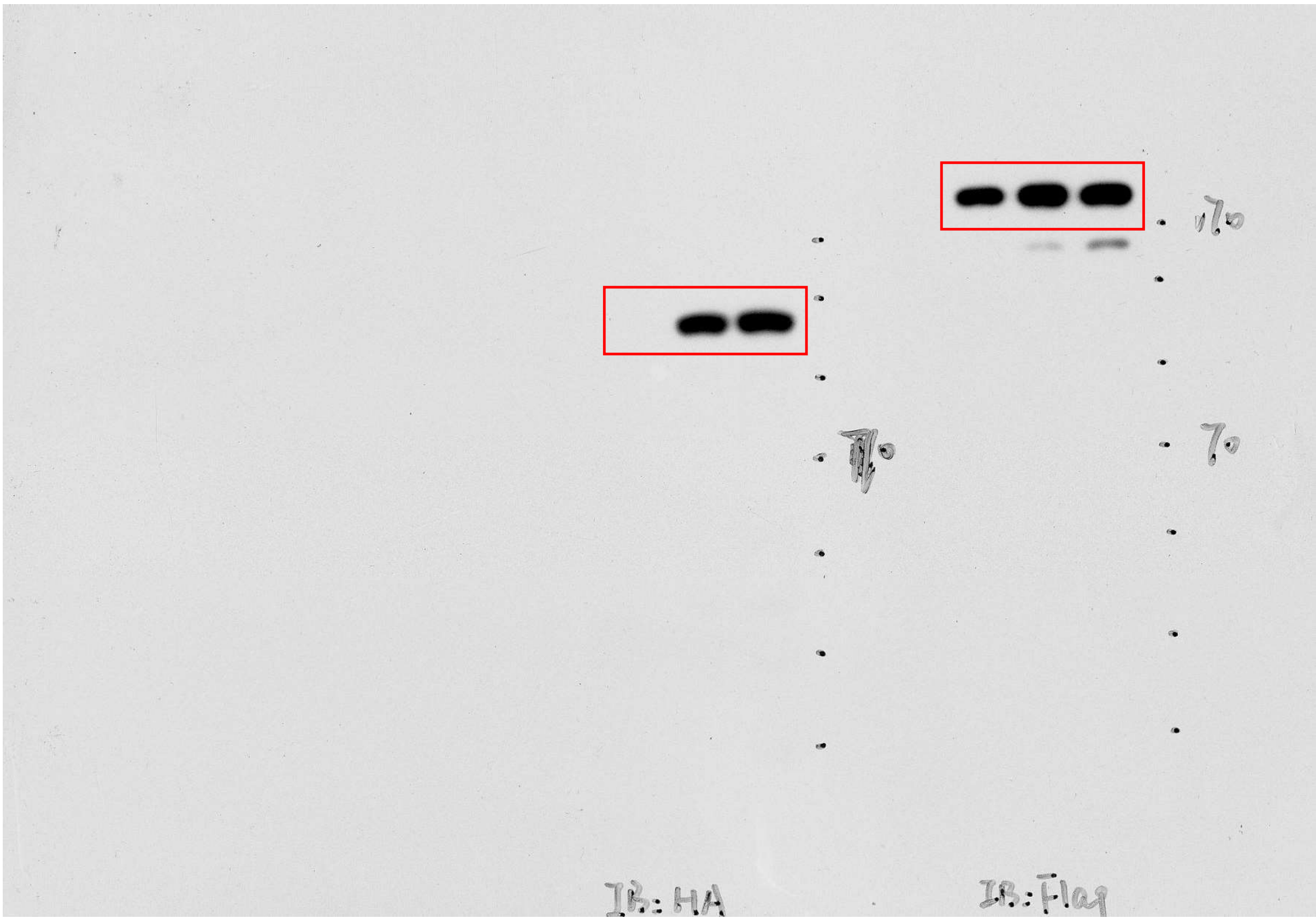

Figure 6 A Right

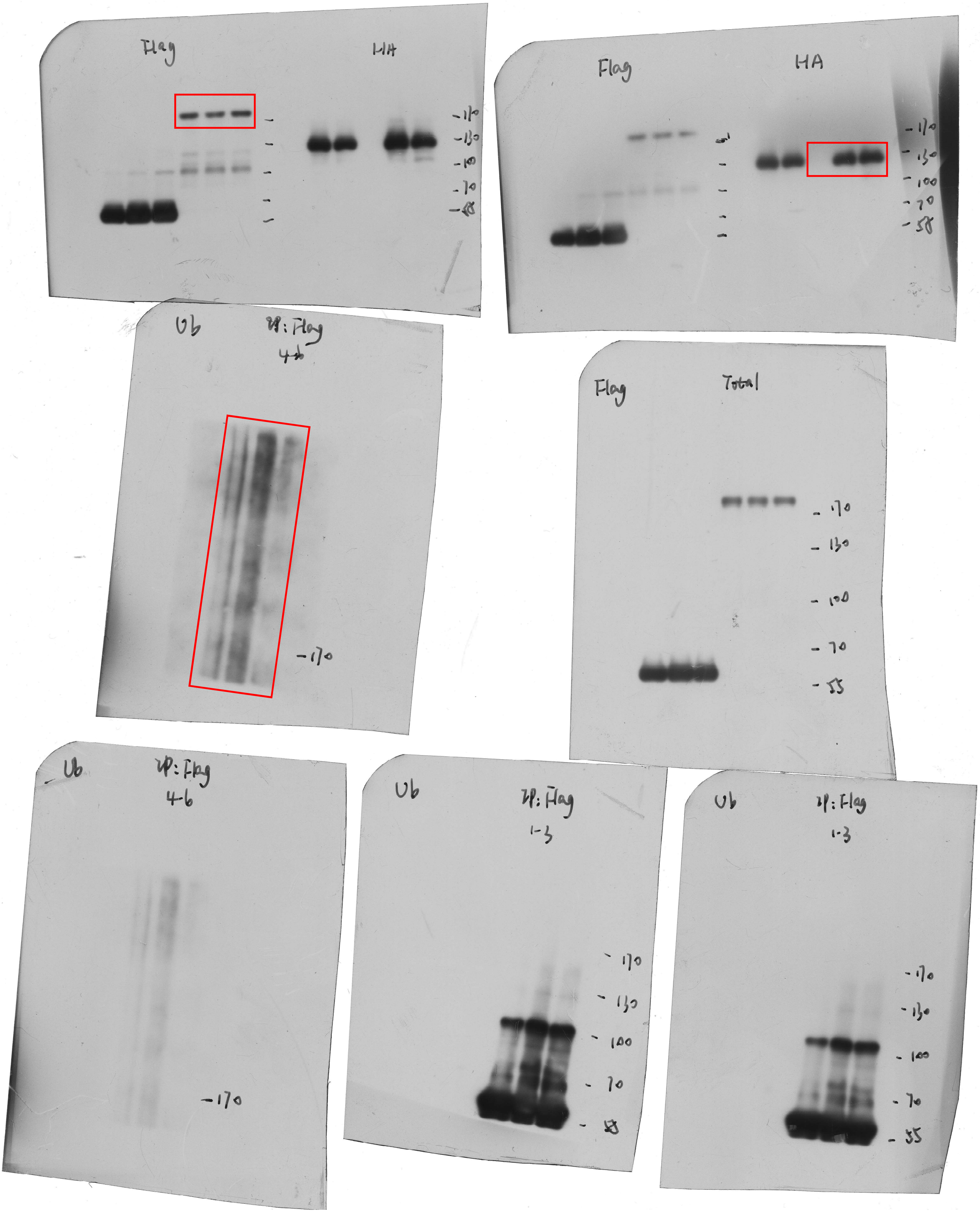

Figure 6 B

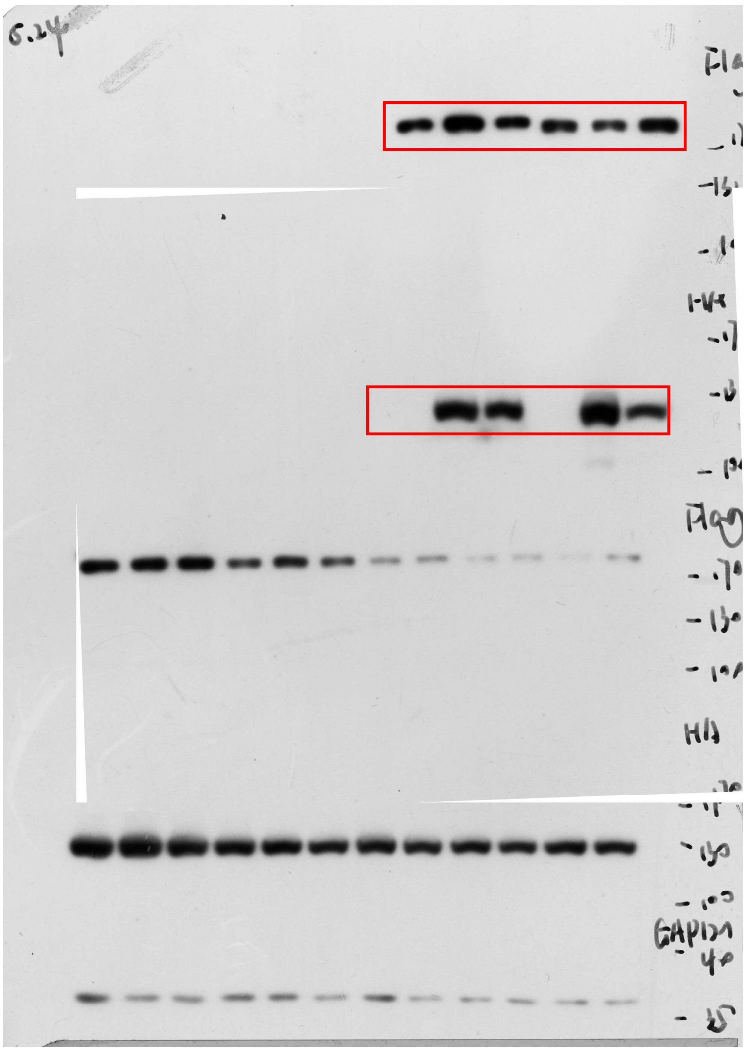

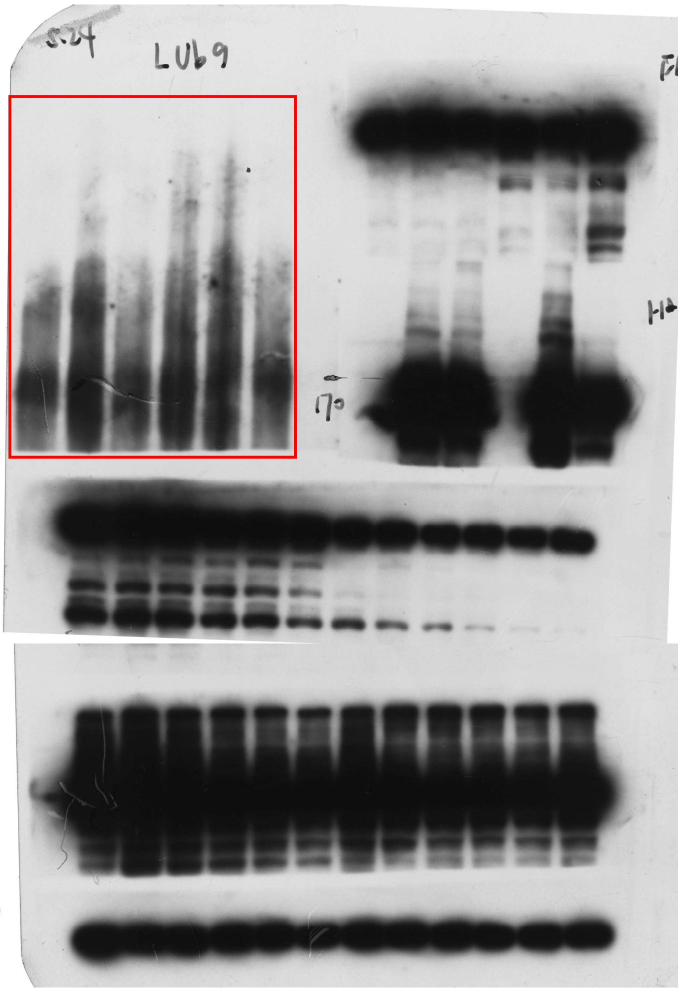

Figure 6 C

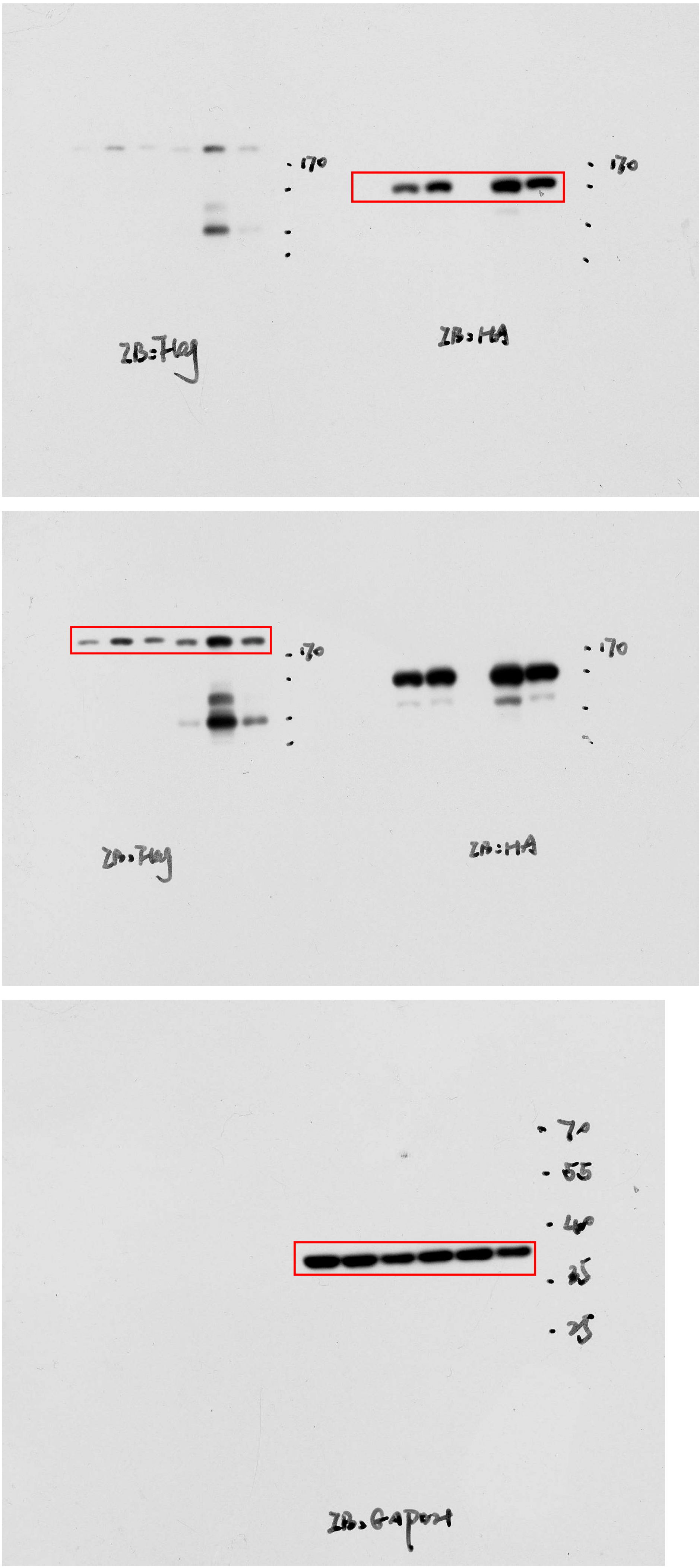

Figure 6D

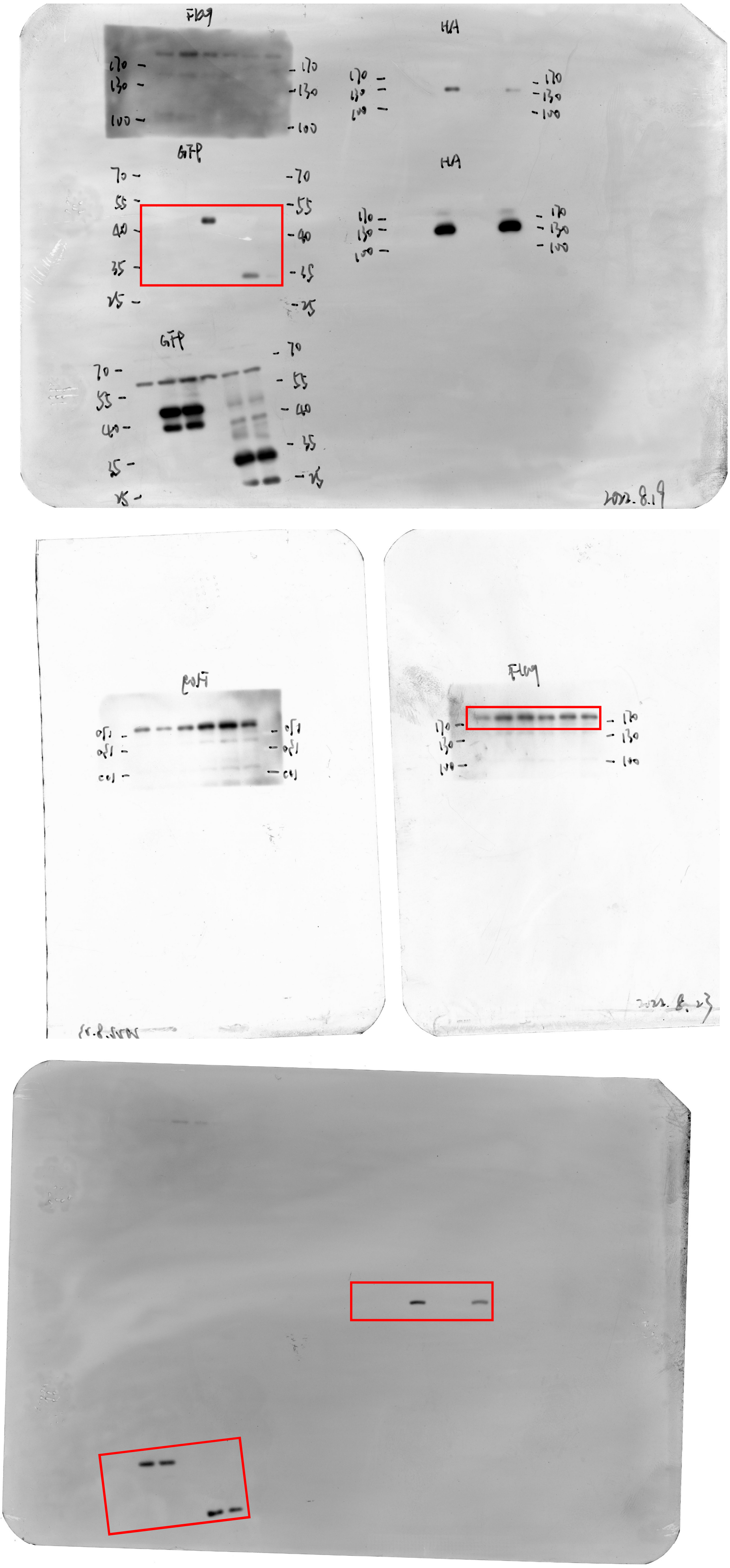

Supplementary Figure 6A

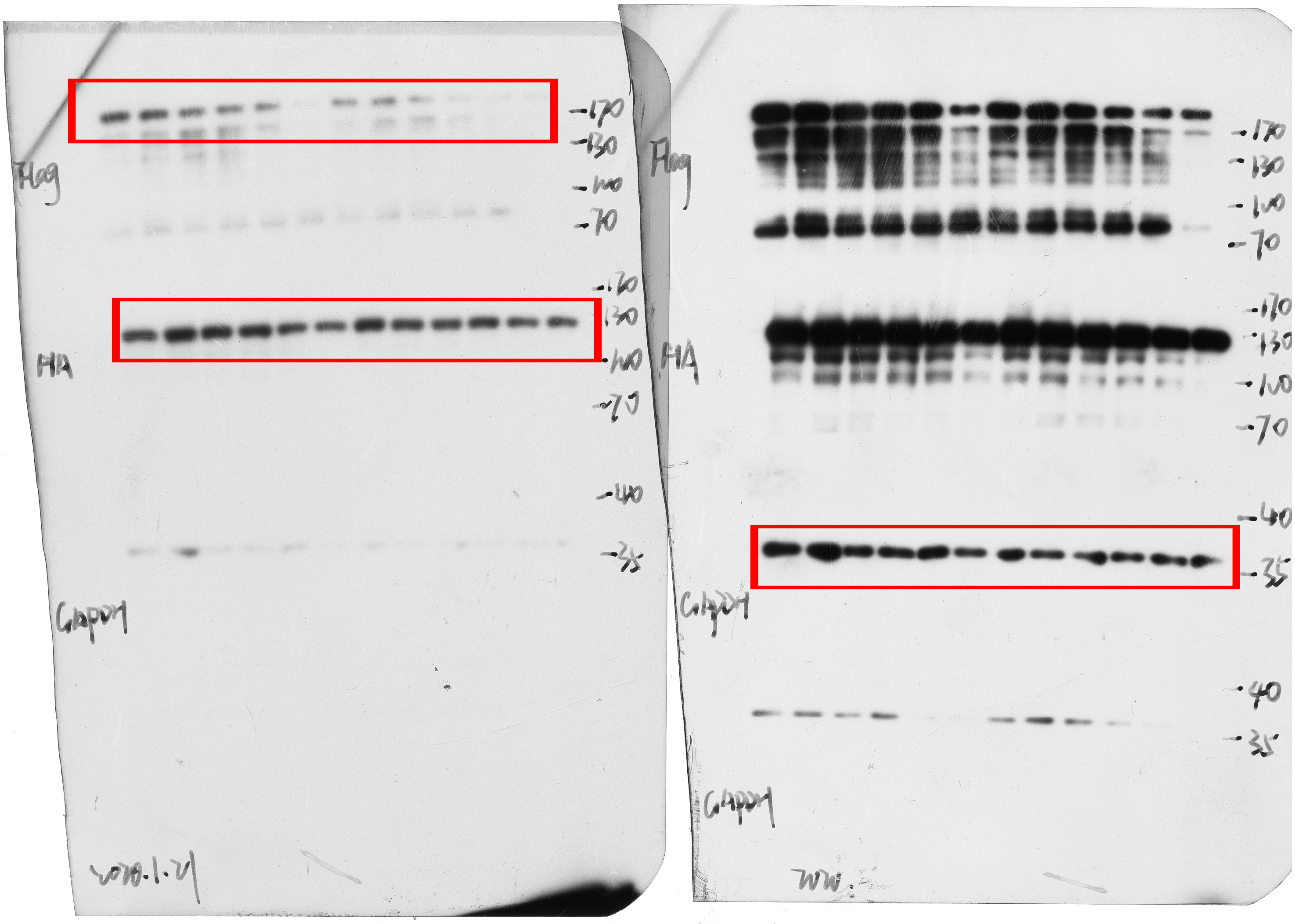

Supplement: Supplementary file 10 — Full-sized immunoblotting films [file 41420_2024_2147_MOESM10_ESM.pdf]
